# Single‐cell sequencing unveils the impact of aging on the progenitor cell diversity in the telencephalon of the female killifish *N. furzeri*

**DOI:** 10.1111/acel.14251

**Published:** 2024-07-01

**Authors:** Rajagopal Ayana, Caroline Zandecki, Jolien Van houcke, Valerie Mariën, Eve Seuntjens, Lutgarde Arckens

**Affiliations:** ^1^ KU Leuven, Leuven Brain Institute, Department of Biology, Animal Physiology and Neurobiology Section Laboratory of Neuroplasticity and Neuroproteomics Leuven Belgium; ^2^ KU Leuven, Leuven Brain Institute, Department of Biology, Animal Physiology and Neurobiology section Laboratory of Developmental Neurobiology Leuven Belgium; ^3^ Leuven Institute for Single‐Cell Omics Leuven Belgium; ^4^ KU Leuven Brain Institute Leuven Belgium

**Keywords:** aging, killifish, neurogenesis, progenitor cells, single‐cell sequencing, telencephalon

## Abstract

The African turquoise killifish (*Nothobranchius furzeri*) combines a short lifespan with spontaneous age‐associated loss of neuro‐regenerative capacity, an intriguing trait atypical for a teleost. The impact of aging on the cellular composition of the adult stem cell niches, leading to this dramatic decline in the postnatal neuro‐ and gliogenesis, remains elusive. Single‐cell RNA sequencing of the telencephalon of young adult female killifish of the short‐lived GRZ‐AD strain unveiled progenitors of glial and non‐glial nature, different excitatory and inhibitory neuron subtypes, as well as non‐neural cell types. Sub‐clustering of the progenitors identified four radial glia (RG) cell types, two non‐glial progenitor (NGP) and four intermediate (intercell) cell states. Two astroglia‐like, one ependymal, and one neuroepithelial‐like (NE) RG subtype were found at different locations in the forebrain in line with their role, while proliferative, active NGPs were spread throughout. Lineage inference pointed to NE‐RG and NGPs as start and intercessor populations for glio‐ and neurogenesis. Upon aging, single‐cell RNA sequencing revealed major perturbations in the proportions of the astroglia and intercell states, and in the molecular signatures of specific subtypes, including altered MAPK, mTOR, Notch, and Wnt pathways. This cell catalog of the young regeneration‐competent killifish telencephalon, combined with the evidence for aging‐related transcriptomic changes, presents a useful resource to understand the molecular basis of age‐dependent neuroplasticity. This data is also available through an online database (killifishbrain_scseq).

## Graphical abstract

We combined single‐cell and long‐read RNA‐sequencing to unravel the progenitors of glial and non‐glial nature, different excitatory and inhibitory neuron subtypes, as well as non‐neural cell types in the young African turquoise killifish telencephalon. This cell‐type catalog of the young regeneration‐competent killifish telencephalon, combined with the evidence for aging‐related transcriptomic alterations in the progenitor populations, presents a useful resource to understand the molecular basis of age‐dependent brain plasticity.

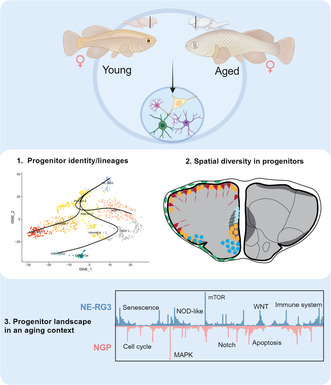

**Abbreviations**
Astro: astroglia‐like
DAPI: 4′,6‐diamidino‐2‐fenylindool
DIV: dividing
EPD: ependymal
GAM: generalized additive model
GO: geneontology
HCR: hybridization chain reaction
IHC: immunohistochemistry
IPC: intermediate progenitor cell
MG: microglia
NC: neuronal cell
NE: neuroepithelial‐like
NFINgb: *N. furzeri* Information Network Genome Browser
NGP: non‐glialprogenitor
NSC: neural stem cell
OD: oligodendrocyte
PBS: phosphate‐buffered saline
PC: progenitor cell
PFA: paraformaldehyde
QC: quality check
RG: radial glia
RT: room temperature
SGZ: sub‐granular zone
SMRT: single‐molecule real‐time
TNB: Tris‐NaCl blocking buffer
TSNE: T‐distributed stochistic neighbor embedding
V‐SVZ: ventricular‐subventricular zone
VZ: ventricular zone

## 1 INTRODUCTION

Adult neurogenesis is a complex biological process involving the production and integration of new neurons into the existing neural network. It has been established that postnatal neurogenesis in vertebrates takes place in specific niches in the brain, and the extent of the process varies from species to species (Chapouton et al., 2007; Zupanc, 2006). In mammals, neurogenesis is largely confined to two neurogenic niches namely, the sub‐granular zone (SGZ) of the hippocampal dentate gyrus and the ventricular‐subventricular zone (V‐SVZ) of the lateral ventricles, albeit noncanonical sites do exist (Ayana et al., 2017; Feliciano et al., 2015; Lim & Alvarez‐Buylla, 2016). Neurogenesis in the mammalian brain is naturally limited and continues to decline upon aging, which is the number one risk factor for neurodegenerative disease (Apple et al., 2017). Aging alters cell–cell communication (the microenvironment) of stem cells, leading to inflammaging and reduced neurogenesis, differentiation, and integration of newborn neurons (Edelmann et al., 2013; Nicaise et al., 2020; Van houcke, Mariën, Zandecki, Seuntjens et al., 2021; Van houcke, Mariën, Zandecki, Vanhunsel et al., 2021). Urodeles and teleost fish are (re)generation‐competent throughout life (Valenzano et al., 2015). The telencephalon of teleosts is anatomically divided into a dorsal pallium and a ventral subpallium, akin to mammals. The neurogenic zones of the dorso‐lateral pallium are considered the equivalent of the mammalian SGZ, while that of the subpallium is homologous to the V‐SVZ (Adolf et al., 2006). The teleost telencephalon develops by eversion or outward folding so that the ventricle surrounds the brain parenchyma and the proliferative ventricular zones (VZ) line the border of each hemisphere (Schmidt et al., 2013). In zebrafish, distinct proliferative zones differ in the rate of development of new cells (Schmidt et al., 2013), and the apical radial glia (RG) form the primary progenitor population (Dirian et al., 2014). Particularly, RG, whose cell bodies are located close to the ventricle, have long processes that extend toward the pial surface of the telencephalon, where they terminate on the walls of blood vessels or at the pial surface (Than‐Trong & Bally‐Cuif, 2015). Zebrafish RG occasionally divide and are the equivalent of adult neural stem cells (NSCs) in mouse neurogenic zones. Rodent GFAP^+^ NSCs are mostly quiescent and give rise to a proliferative, active NSC or a transiently amplifying progenitor type by asymmetric division (Daynac & Petritsch, 2017). Zebrafish neuroepithelial cells, which are neural progenitor cells inherited from embryogenesis, help in the constant supply of new RG in the VZ, that later enter quiescence and serve as neurogenic NSCs (Than‐Trong & Bally‐Cuif, 2015).

The relatively long lifespan of model species such as mouse, zebrafish, and goldfish has been a major disadvantage to study the specifics of age‐related neural decline in detail. The African turquoise killifish, *Nothobranchius furzeri*, is an excellent aging model to fill this gap since it combines a short lifespan with the expression of mammalian‐like aging hallmarks (Bergmans et al., 2023; de Bakker & Valenzano, 2023; Hu & Brunet, 2018). In particular, the GRZ‐AD inbred strain is the shortest‐lived of all killifish strains, with a median lifespan of only 4–6 months in laboratory conditions (Polačik et al., 2016; Reichwald et al., 2015; Valenzano et al., 2015). *N. furzeri* shares more than 90% of orthologous genes with zebrafish, and over 70% with mammalian genomes (Howe et al., 2013; Van houcke, Mariën, Zandecki, Vanhunsel et al., 2021). The killifish CNS is prone to aging, with typical age‐associated hallmarks including lipofuscin accumulation, impaired protein homeostasis, mitochondrial dysfunction, reactive gliosis, reduced neurogenesis, and spontaneous neurodegeneration, eventually leading to clear impairments in locomotor, learning, and memory function (Bergmans et al., 2023; Mariën, Piskin et al., 2024). Upon injury, the brain of young killifish repopulates with new neurons whereas in aged fish permanent glial scarring goes hand in hand with a much‐reduced regenerative capacity (Van houcke, Mariën, Zandecki, Vanhunsel et al., 2021).

Interestingly, previous work on killifish revealed that it is not RG but another highly proliferative population termed non‐glial progenitors (NGPs) that actively drives the neuro‐differentiation process in the dorsal telencephalon (Coolen et al., 2020). These NGPs were observed in both juvenile and adult killifish at the apical surface, dispersed in between the RG. They are characterized by a more immature morphology and express markers of division, including PCNA, and neural progenitor markers NESTIN, MSI1, while being devoid of pan‐glia/RG markers like GLUL, FABP7, or GFAP (Coolen et al., 2020). Based on their proliferative character, NGPs could be similar to mammalian intermediate progenitor cells (IPC) which are known for amplifying the stem cell progeny before differentiating into neurons or glia. However, juvenile killifish seem to be full of self‐renewal and neurogenic potential, resembling early neuroepithelial progenitors. They have an apical domain contacting the ventricle, and express NESTIN, which are characteristics of neuroepithelial cells rather than IPCs (Coolen et al., 2020).

What kind of progenitor subtypes exactly reside in the killifish telencephalon and how aging affects their cellular and molecular profiles remains unexplored. In‐depth knowledge about the full cell diversity in young and aged killifish, including glial and non‐glial progenitor cells, and their full molecular signature is imperative to understand the cellular and molecular underpinnings of aging‐induced deficits in neurogenic potential. Here, we took the first steps to investigate the cellular heterogeneity of the telencephalon of the fast‐aging *N. furzeri* GRZ‐AD strain. We utilized single‐cell RNA sequencing of female 6‐week‐young and 18‐week‐old telencephalon samples to reveal the cellular diversity with a special focus on progenitor diversity. For the young age, we could establish transcriptomes of 9616 cells, which represent known and novel killifish brain cell types including NGPs, distinctive RG, neuronal (NC), and non‐neuronal subtypes in the adult brain. Iterative sub‐clustering followed by lineage inference analyses of the progenitor cells (PCs) delineated three trajectories including four RG subtypes, two NGP and four intermediate cell states marking the transition between progenitors. The spatial setting for the progenitor subtypes revealed differential cellular organization in the neurogenic niches. Aging (9767 cells) induced extensive changes in the molecular profiles of root cell types in the lineage analysis; NGP and neuroepithelial‐like RG. This unique and detailed map (~20,000 cells) of young versus aged killifish neural cell diversity creates a firm basis for future investigations of age‐dependent cell‐type driven mechanisms of neurogenesis upon injury and disease (de Bakker & Valenzano, 2023; Van houcke, Mariën, Zandecki, Seuntjens et al., 2021; Van houcke, Mariën, Zandecki, Vanhunsel et al., 2021).

## 2 RESULTS

### 2.1 Single‐cell sequencing identifies main cell types in the adult killifish telencephalon

**FIGURE 1:**
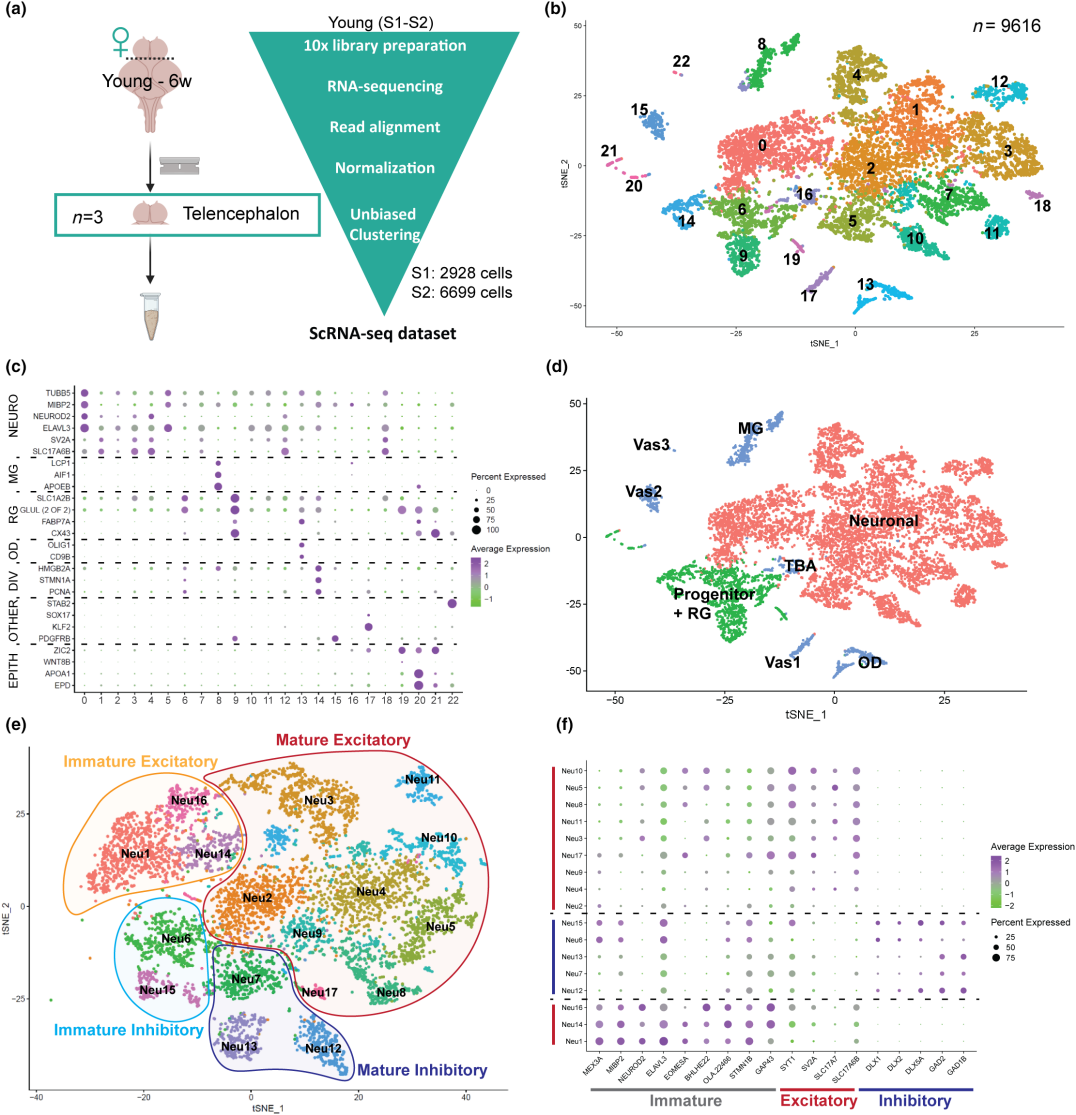
Cell type identification and categorization in the adult killifish telencephalon. (a) Seurat‐based single‐cell analysis strategy for adult telencephalon samples. (b) TSNE Plot shows 23 identified cell clusters (Resolution 0.6). (c) Dot plot shows markers for all identified cell types within the adult telencephalon. The size of the dot indicates the percentage of cells expressing the gene (0–100%), the color indicates the expression level. Full list of gene markers that identify 23 cell types (Table S2). (d) TSNE plot shows the broad cell categories; color‐coded for Neuronal cells: pink, Progenitor cells: green, Other cells: blue. (e) TSNE Plot shows cell type composition of neuron subtypes (Neu1‐17). (f) Markers for diverse neurons and ascertaining differential trajectories for immature/mature, excitatory vs. inhibitory neurons in the killifish telencephalon. DIV, dividing; EPITH, epithelial; MG, microglia; NEURO, neuronal; OPC/OD, oligodendrocyte progenitor cell/oligodendrocyte; RG, radial glia; TBA, to be annotated; Vas, vasculature.

We performed single‐cell RNA sequencing using the 10X Chromium method (Figure 1a). Using an optimized cell dissociation protocol we obtained high viability cell suspensions (>97%) of two 6‐week‐old young adult female killifish telencephalon samples (Mariën, Arckens et al.,  2024). Sequencing reads were mapped to the killifish reference genome (Nfu_20140502). Mapping statistics showed that only 45% of the single‐cell reads confidently mapped to the available *N. furzeri* transcriptome (available through reference gene annotations). To improve the read mapping, we additionally performed Single Molecule, Real‐Time (SMRT) Sequencing, and Iso‐Seq analysis on whole telencephalon RNA of 6‐week‐old killifish telencephalon. This rendered full‐length cDNA sequences (no assembly required) to characterize full‐length transcripts and isoforms across the entire transcriptome. Thus, we attained better genome and transcriptome coverage and increased the sequenced read mapping from 45% to 69% (Table S1). We recovered 9627 single cells after integrating the two independent samples, with an average of 25,800 mean reads per cell and 828 genes per cell (Table S1). The two samples clustered well with minimal batch effects upon integration (Figure 1b, Figure S1a,b). Cell quality check and clustering analysis were performed using Seurat. After removing cells with minimum and maximum thresholds for nUMI, nGene, ribosomal, and mitochondrial RNA genes, we obtained 9616 high‐quality cells (Figure S1b–h). The number of principal components or dimensions for further analyses was limited to 30 based on the standard deviation between dimensions (Figure S1f). We further performed scaling and normalization, and clustered the cells using a dimensionality reduction method, t‐distributed stochastic neighbor embedding (TSNE), which resulted in 23 clusters (0–22, Figure 1b).

Annotation of clusters was performed using known marker genes for cell types in vertebrates (Figure 1c,d, Table S2). On this coarse level, we first annotated neuronal clusters, progenitors, and radial glia‐like clusters versus the remaining clusters (Figure 1d). As expected, most clusters had a clear neuronal signature [NEURO], such as beta‐tubulin (TUBB5), the neuronal bHLH transcription factor NEUROD2, the neuronal marker gene ELAVL3, the synaptic marker SV2A, or the glutamate transporter SLC17A6B. Other clusters included microglia [MG], marked by L‐plastin (LCP1), IBA1 (AIF1), and apolipoproteinE (APOEB); oligodendrocytes [OD], marked by OLIG1 and the surface marker CD9 (CD9B); endothelium, marked by platelet‐derived growth factor receptor beta (PDGFRB); red blood cells, marked by the transcription factors KLF2 and SOX17; phagocytosing cells marked by STAB2 (Vas1‐3 in Figure 1d). One cluster did not express clear markers and could not be annotated [TBA]. Radial glia‐like cells [RG] were identified based on expression of GLUL (Glutathione synthetase 2), the amino‐acid transporter SLC1A2B (EAAT2 and GLT), the fatty‐acid binding protein FABP7A (BLBP), and the gap‐junction protein CX43. Some of these clusters also expressed epithelial markers such as ZIC2 and WNT8B or ependymal markers such as apolipoprotein APOA1 and ependymin (EPD). The histone‐binding protein HMGB2A, the mitotic cell cycle marker Stathmin (STMN1A), and the proliferation marker PCNA were co‐expressed in clusters of dividing cells [DIV].

### 2.2 Neuronal cell type diversity

To get a more refined view on neuronal diversity, we subclustered all clusters annotated as [Neuronal] in Figure 1d. This yielded 17 clusters (Neu1‐17 in Figure 1e,f), which we could subdivide into 12 clusters with an excitatory signature, and five clusters with an inhibitory signature. Within each signature, there were immature states, marked by a higher expression of markers of newborn neurons, such as ELAVL3 and GAP43, and transcription factors EOMESA and BHLHE22 (pallial), DLX1/2 (subpallial, inhibitory), and the stemness marker MEX3A. Mature character was marked by expression of synaptic proteins (SYT1 and SV2A), vesicular glutamate transporters SLC17A7 and SLC17A6B (excitatory), or GABA‐synthetizing enzymes GAD2 and GAD1B (inhibitory) (Figure 1f, Table S3). These results showed that the killifish telencephalon contained relatively more excitatory neurons, and different subtypes of excitatory and inhibitory neurons. Within the excitatory neurons, we observed two different lineages, characterized by differential EOMESA expression: a low‐expressing group of clusters (immature 16 and mature 3 and 11) and a high‐expressing group (immature 1, 14 and mature 5, 8, 10, 17) (Figure 1f). Overall, clusters 2, 4, and 9 only had mildly elevated levels of marker genes, indicating an overall transcriptional downregulation upon differentiation.

### 2.3 Progenitor cell type diversity

**FIGURE 2:**
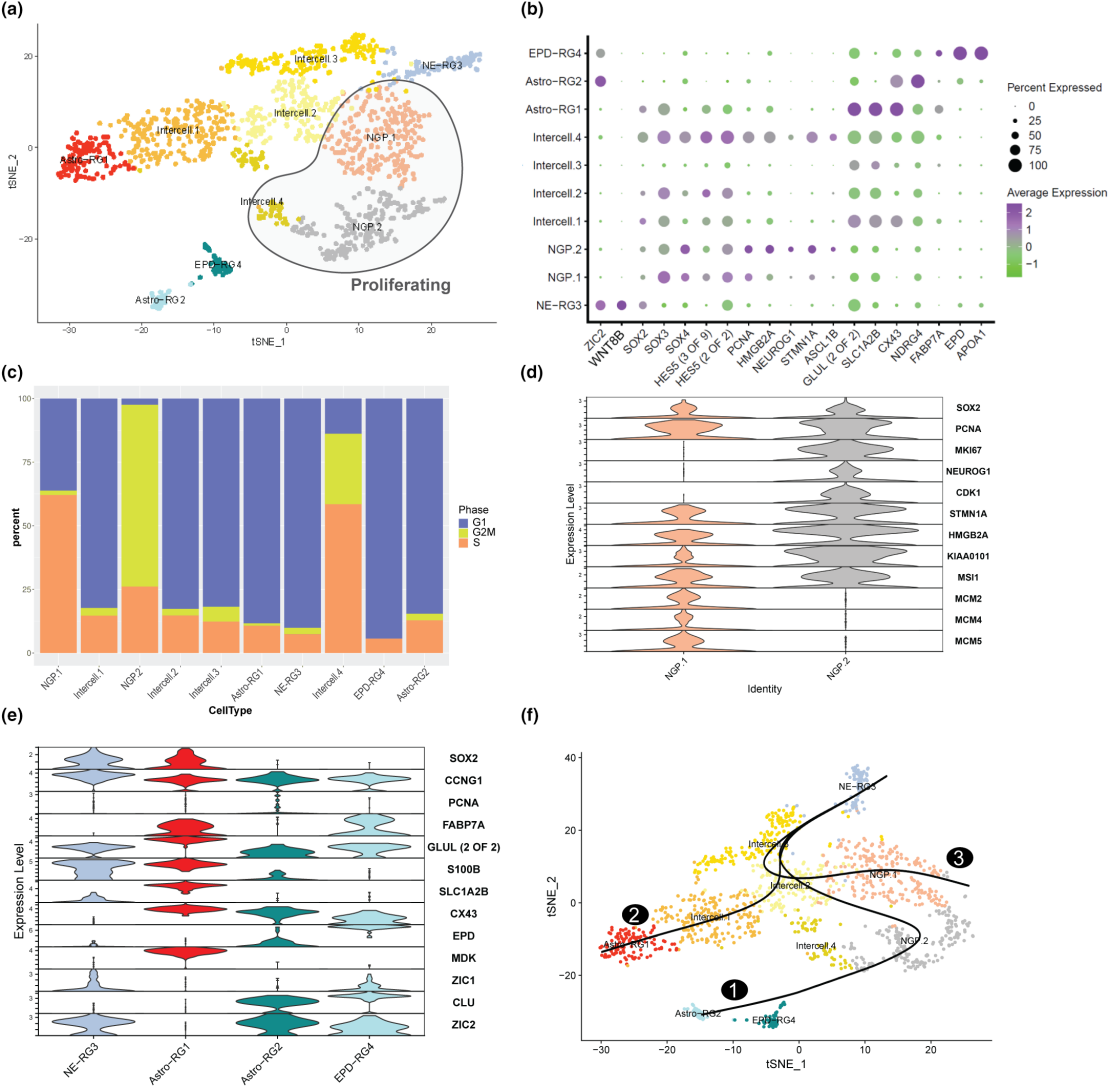
Identification of progenitors and relationships. (a) Iterative clustering of progenitor cells shows 10 cell subtypes. (b) Dot plot shows markers specific to progenitor subtypes. The size of the dot indicates the percentage of cells expressing the gene (0%–100%), the color indicates the expression level. (c) Cell cycle phase proportion differences between main progenitor subtypes indicating their level of activity/quiescence. (d) Gene expression differences in cell‐cycle gene sets between the two NGP cell states. (e) Distinct markers of the four RG subtypes. (f) Slingshot‐based lineage inference analysis shows three divergent trajectories starting with NE‐RG3. Astro‐RG1, Astroglia‐like Radial glia 1; Astro‐RG2, Astroglia‐like Radial glia 2; EPD‐RG4, ependymal radial glia 4; Inter‐cell, intermediate cells; NE‐RG3, neuroepithelial radial glia 3; NGP, non‐glial progenitor.

In general, teleost fish can generate neurons over the entire lifespan in numerous brain regions including the telencephalon. In zebrafish, these neurons are generated from activated radial glia and fast‐cycling progenitors (Dirian et al., 2014). Recent evidence showed that besides radial glia, killifish have an actively dividing non‐glial progenitor cell type in the outer layer of the telencephalon (Coolen et al., 2020). Here, we wanted to profile the diversity of proliferating and radial glia‐like cell types in depth. Expression of GLUL (glial) and PCNA (proliferating) progenitor markers were used to extract all PC subtypes for further analyses (Bradley et al., 2019; Malatesta et al., 2008) (Figure S2a, Table S4). After the coarse definition (Figure 1d), we subclustered these presumptive progenitor cells (1303 cells) and found 10 clusters (resolution 0.8) that we could annotate in a biologically meaningful manner (Figure 2a,b).

Three clusters (NGP.1, NGP.2, and Intercell.4) showed a clear proliferative character, all of them lacked glial markers, which is in line with the non‐glial progenitor (NGP) described before. NGPs display two states, one (NGP.1) of which the cells were predominantly in G1 or S‐phase (Figure 2c), and expressed mini chromosome maintenance (MCM) genes, that are essential for DNA replication (Forsburg, 2004) (Figure 2d), while most of the NGP.2 cells were in G2/M phase (Figure 2c) and expressed cell cycle genes CDK1 and 2 (Figure 2d). The majority of Intercell.4 cluster cells were in S or G2/M phase (Figure 2c), and these cells also expressed low levels of glial markers, suggesting this cluster is a transition cell state in the path of gliogenesis.

Four clusters had a radial glial (RG) signature, expressing GLUL, SLC1A2B, FABP7A, and CX43 (Figure 2b,e). SLC1A2B and CX43 were used to identify mature astroglia (Astro‐RG1, Astro‐RG2), which have been similarly annotated in mammals, and in juvenile killifish (Coolen et al., 2020) (Figure 2B). The density of cells expressing CX43 was similarly high in both astroglial subtypes, whereas CX43 expression was lower or absent in other RG subtypes (Figure 2b, Figure S2b). Genes exclusive to Astro‐RG1 included neurotransmitter transporters SLC1A2B and SLC6A11, metabotropic glutamate receptors GRM3 and GRM7, and GABA receptor GABBR1. Astro‐RG2 expressed genes such as ATP1A2B, ATP1B1, SLC7A3A, and astrocyte‐enriched markers like SLC3A2 and SLC7A5 that are involved in amino acid transmembrane transport (Batiuk et al., 2020; Cuevas‐Diaz Duran et al., 2019) (Figure S2b).

Next to Astro‐RG1 and Astro‐RG2, we identified two other RG types; neuroepithelial RG cells (NE‐RG3) and ependymo‐glial cells (EPD‐RG4). NE‐RG3 expressed known (neuro)epithelial cell markers such as ZIC1, ZIC2, and KRT18 (Figure 2b,e, Table S4). Additionally, the expression of RG self‐renewal associated factors such as SOX2, WNT8B, EMX2, and SIX3 were also found to be upregulated in NE‐RG3 (Sanek et al., 2009) (Figure 2b, Table S4). Due to an everted telencephalon in teleosts, the tela choroidea or ependymal layer surrounds the ventricular surface. In other teleosts, the ependyma contains mainly RG‐like cells and neuroepithelial cells located at the ventricular surface (Jurisch‐Yaksi et al., 2020). We found one RG subtype linked to ependyma, EPD‐RG4, which expresses ependymin (EPD), as well as apolipoprotein genes APOA1, APOA2, APOEB, and CLU (APOJ) (Figure 2b,e, Table S4). We propose that EPD‐RG4 resembles a non‐ciliated ependymo‐glial cell type as this cluster is devoid of ciliary filament‐associated genes (D'Gama et al., 2021). Taken together, we found distinct glial subtypes within the telencephalon that have epithelial, astroglial, or ependymal signatures.

### 2.4 NE‐RG3 have features of quiescent multipotent progenitors

To identify the root cluster of neurogenesis, we analyzed the expression of highly conserved molecular players (transcription factors) and growth factor pathway molecules. Transcription factors associated with stemness (Stevanovic et al., 2021, 2023), such as SOX2 and SOX3 were relatively highly expressed in Astro‐RG1 and NE‐RG3, while all SOX transcription factors were clearly expressed in NGP.1 and NGP.2 (Figure 2b,d). Cooperation between Notch and BMP pathways is necessary to control stem cell quiescence, and most neural stem cells are known to be quiescent during adulthood (Dirian et al., 2014; Zhang et al., 2021). Therefore, we probed for Notch and BMP signaling molecules and downstream effectors such as HES and ID genes within the prominent progenitor cell types NE‐RG3, NGP.1/2, and Astro‐RG1/2 (Bansod et al., 2017; Kageyama et al., 2008) (Figure S2c). In our data, only NE‐RG3 expressed HES1 (her6), HES4 (her9), and HES5 (her4/2), as well as all ID1–4 genes. This pattern suggests high levels of Notch and BMP signaling, and a quiescent stem cell phenotype. Intriguingly, the other progenitor cell clusters expressed a particular subset of HES and ID genes; Astro‐RG1, and both NGP clusters had high levels of HES1, HES5, ID1, and ID4, whereas Astro‐RG2 and EPD‐RG4 had clear HES4 and ID3 expression, but little or no HES5, ID2, or ID4 expression. Astro‐RG2 also expressed ID1 (Figure S2c). In juvenile killifish, it has previously been shown that RGs enter NOTCH3‐mediated quiescence prematurely (Coolen et al., 2020). In the adult telencephalon, NOTCH3 was highly expressed in NE‐RG3 and Astro‐RG1 (Figure S2c). NGPs are known to use the pro‐neural gene NEUROG1 and rely on Notch1 signaling for their maintenance in juvenile fish. In our data, NOTCH1 and NEUROG1 are mainly expressed in NGP.2 although the expression level is low (Figure S2c). Taken together, NE‐RG3 might represent the most plausible root stem cell type, while other PC clusters have maintained a subset of Notch and BMP target genes that might underly different, perhaps region‐dependent functions.

### 2.5 Intercell clusters represent intermediate cell states in a differentiation trajectory

In our dataset, sub‐clustering of PCs revealed several clusters that we named Intercell.1, Intercell.2, Intercell.3, and Intercell.4 (Figure 2a). All four Intercell clusters were found to express HES5 and ID4, which might indicate that they represent intermediate states in a neuro‐ or gliogenic path (Figure 2b, Table S4). Intercell.1–3 displayed reduced expression of typical markers of RG (Astro‐RG1/2, NE‐RG3, and EPD‐RG4) and NGP (Figure 2b). The complete gene list of shared or unique genes can be retrieved (Table S4).

Contrary to zebrafish, NGPs and not RG were recently proposed as the neurogenic progenitor type in the juvenile and adult killifish pallium (Coolen et al., 2020; Kroehne et al., 2011; Rothenaigner et al., 2011; Van houcke, Mariën, Zandecki, Vanhunsel et al., 2021). Whether the progenitor cell subtypes are related to each other and represent different steps in the neurogenic process remains unclear. We performed lineage inference analysis of the progenitor cell subclusters using Slingshot. The neuroepithelial cell signature (Figure 2b,e, Figure S2c) posed NE‐RG3 as the most likely root cluster candidate. Analysis using NE‐RG3 as the root or start cluster revealed three possible lineages, each of which passed through several Intercell clusters to reach diverse terminal cell states (Figure 2f). The first lineage (1) started with NE‐RG3 followed by Intercell.3, NGP.2, and Intercell.4, to give rise to Astro‐RG2 and EPD‐RG4 suggesting EPD‐RG4 and Astro‐RG2 are molecularly similar clusters (Figure 2f). This corroborated the finding that Intercell.4 is gliogenic. The second lineage (2) terminated in the formation of Astro‐RG1 via 3 Intercell clusters (Intercell.3, Intercell.2, and Intercell.1) (Figure 2f). Since the astroglial subtypes (Astro‐RG1/2) are formed via different lineages, a functional difference is expected. A third lineage (3) via Intercell.3 and Intercell.2 to NGP.1 is likely the neurogenic path, as NGP.1 expressed pro‐neural transcription factors.

We further performed Generalized Additive Model (GAM) based analysis to identify lineage‐specific temporally expressed genes or genes that change with pseudotime. Over pseudotime (0–100), we assessed the expression of these genes across the three lineages. The top genes included midkine (MDK), HES5, CX43, and ZIC2 (Figure S2d). The neurotrophic cytokine MDK was lowly expressed initially but increased in lineage 2 within Intercell.3/2/1 to reach its peak value in Astro‐RG1 (Figure S2d). In zebrafish, it has been established that in the telencephalon MDK is mainly restricted to S100B^+^ RG (Lübke et al., 2022) and in our data, we find correlative expression in S100B^+^ Astro‐RG1. In zebrafish, the expression of different HES genes marks radial glial cell populations. We found HES5 to be expressed heavily in all Intercell clusters. Expression was lower in NE‐RG3 and increased in lineages 2 and 3 (Figure S2d). As mentioned before, HES5 expression was even more reduced upon reaching maturation in Astro‐RG2 and remained higher in Astro‐RG1. As expected, CX43 was lowly expressed in NE‐RG3 and NGPs and peaks in terminal clusters Astro‐RG1/2, and their possible precursor clusters Intercell.1 and Intercell.4 in lineage 2 and 1, respectively (Figure S2d). ZIC2 was highly expressed in NE‐RG3 followed by downregulation across lineages up until reappearing in Astro‐RG2 and EPD‐RG4 (Figure S2d). We found ZIC2 upregulated early in pseudotime scale, indicative of an early progenitor marker. In conclusion, trajectory analysis identified a possible neurogenic and two distinct gliogenic routes.

### 2.6 Astroglial, ependymal, and neuroepithelial radial glial cell types have distinct spatial locations

**FIGURE 3:**
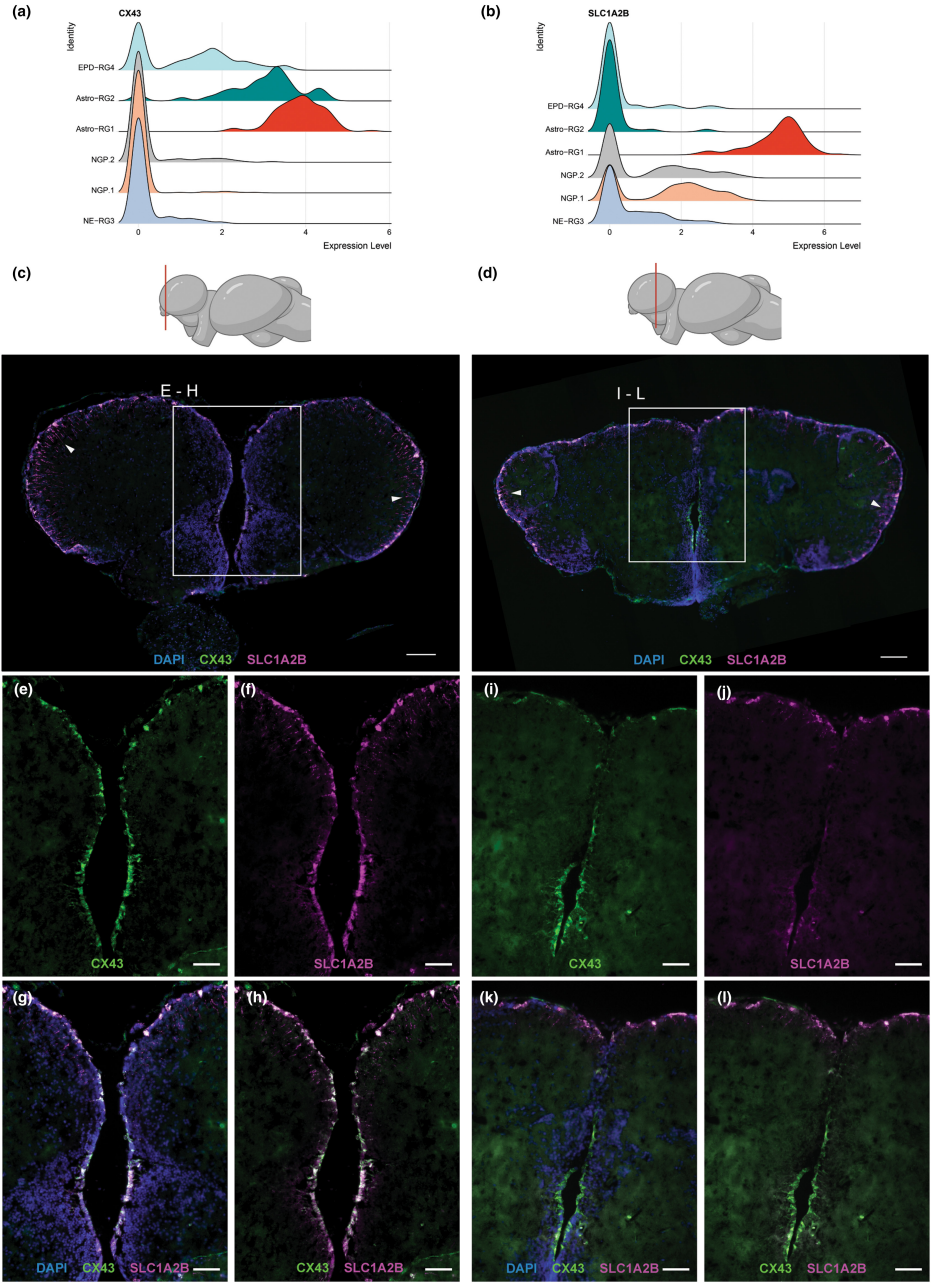
Spatial organization of identified astroglia subtypes: Astro‐RG1 and Astro‐RG2. Ridge plots show the density of cells expressing the astroglial markers (a) CX43 and (b) SLC1A2B, in six main progenitor cell types. The higher the peak, the more cells display a particular expression level in the specific cell type. Multiple peaks indicate differential gene expression within the same cluster. Singular cell peaks at expression 0 are indicative of low/zero expression of the gene in all cells of that cluster. Rostral (c) and caudal (d) coronal sections of the telencephalon, the anterior–posterior position of the section is indicated in red on a lateral side view killifish brain illustration. Fluorescent in situ labeling of SLC1A2B (magenta) and CX43 (green) mRNA expression in combination with a nuclear stain (DAPI, blue) marks Astro‐ RG1 and Astro‐RG2, respectively. For both sections, CX43 and SLC1A2B expression are present in the cells lining the ventricular surface. Also, RG fibers can be identified, extending into the parenchyma; white arrowheads point to zones where they are visible. Scale bar: 100 μm. (e–h) Magnifications of the zone are depicted with a square in the rostral section C. Co‐labeling of SLC1A2B and CX43 is observed in cells lining the ventricular surface and at the conjunction of the two hemispheres, identifying Astro‐RG1 as one of the main cell types at the ventricular surface. Scale bar: 50 μm. (i–l) Magnifications of the zone are depicted with a square in the caudal section D. Co‐labeling of SLC1A2B and CX43 is observed in cells at the dorsal surface (Astro‐RG1). At the conjunction of the two hemispheres, detection of only CX43 mRNA expression is observed, identifying Astro‐RG2 as an astroglial‐like cell type spatially restricted to the ventro‐caudal conjunction of the two hemispheres. Scale bar: 50 μm.

To further investigate the functional diversity between the two astroglia cell types RG1 and RG2, we examined their spatial organization via hybridization chain reaction (HCR) for the CX43 and SLC1A2B markers (Figure 3a,b). Visualization of mRNA on rostral and caudal coronal sections of the telencephalon revealed the spatially distinct location of Astro‐RG1 (CX43^+^ and SLC1A2B^+^) and Astro‐RG2 (CX43^+^ and SLC1A2B^−^) (Figure 3c,d). Astro‐RG1 were particularly present at the outer border of the everted telencephalon, lining the ventricular surface. More rostrally, an abundance of Astro‐RG1 was detected at the lateral, medial, and dorsal surface, almost completely surrounding the two telencephalic hemispheres (Figure 3c,e–h). Caudally, Astro‐RG1 was also lining the dorsal and lateral surface (Figure 3d), whereas Astro‐RG2 was exclusively present at the ventro‐medial conjunction of the two hemispheres (Figure 3d,i–l). The detection of SLC1A2B and CX43 transcripts in glial fibers extending into the parenchyma further confirmed the astroglia‐like properties (Sakers et al., 2017) of these two RG populations (Figure 3c,d). Astro‐RG1 and Astro‐RG2 thus represented two spatially separated cell populations, which might explain their different transcriptional profile.

**FIGURE 4:**
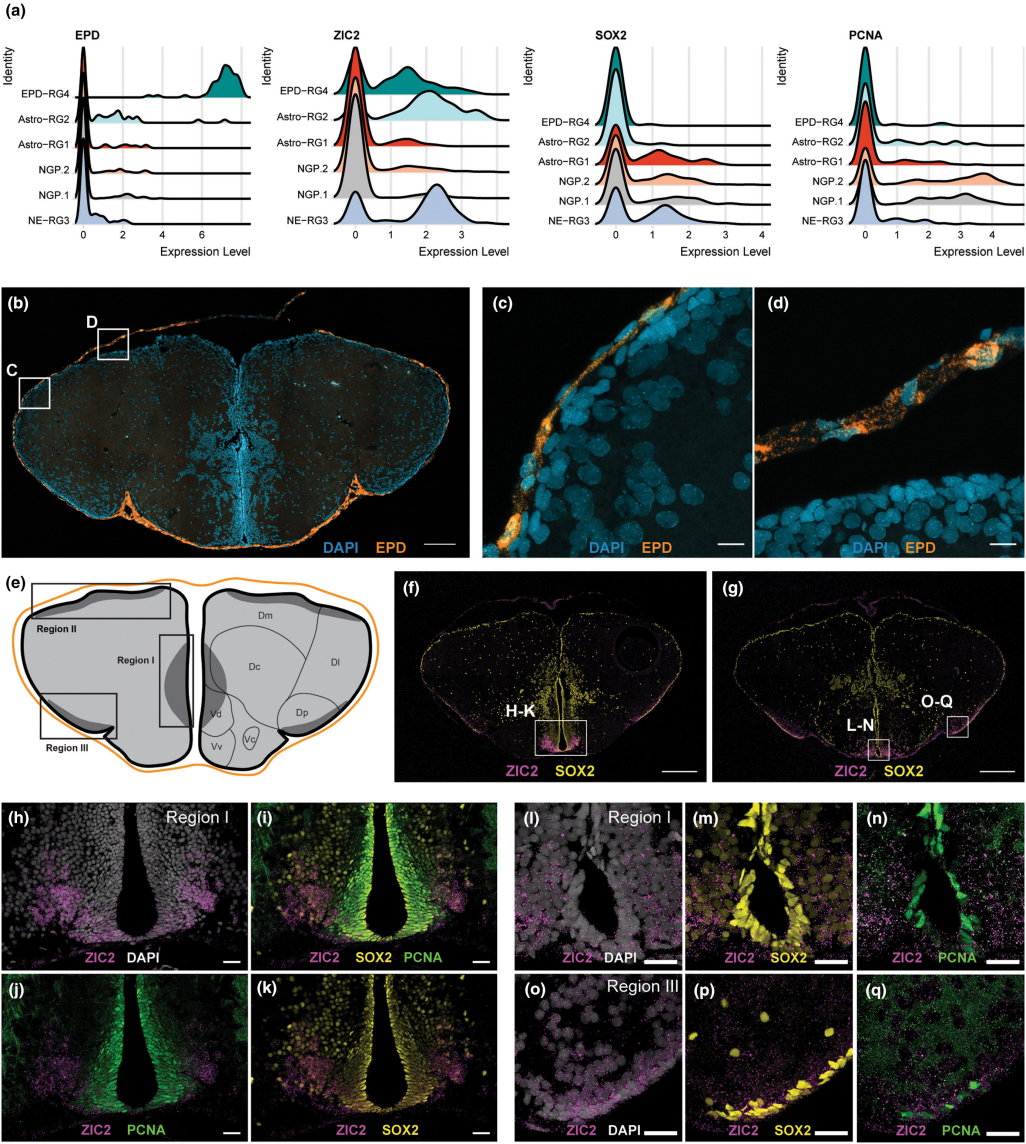
Ependymo‐glial cells (EPD‐RG4) in the tela choroidea. (a) The ridge plot shows the density of cells expressing the EPD‐RG4 marker EPD, the NE‐RG3 marker ZIC2, the progenitor marker SOX2 and the proliferation marker PCNA, in six main PC subtypes. (b) The coronal section of the telencephalon shows fluorescent in situ labeling of EPD (orange) in combination with a nuclear stain (DAPI, blue). The fluorescent signal can be observed surrounding the ventricular surface. Scale bar: 100 μm. (c, d) Magnification of zones depicted with squares in (b). Fluorescent labeling of EPD mRNA expression (orange) is clearly restricted to the tela choroidea. Scale bar: 10 μm. (e) Illustration of the tela choroidea (orange) surrounding the everted telencephalon as visible on a coronal brain section. The three neurogenic niches (I–III; dark grey) are indicated. Region I is located at the ventral conjunction of the hemispheres, regions II and III are located at the ventricular surface, at the medial to lateral and posterior zone of the dorsal pallium, respectively. The dorsal (D) and ventral (V) subdomains present in the telencephalon are annotated. (f, g) Coronal sections around the mid‐anterior–posterior level show fluorescent in situ labeling of ZIC2 (magenta) in combination with immuno‐histochemical staining for PCNA (green) and SOX2 (yellow). Scale bar: 200 μm. (h–q) Magnification of the boxed areas in f and g. Scale bar: 20 μm. Dc, central zone of D; Dl, lateral zone of D; Dm, medial zone of D; Dp, posterior zone of D; Vc, central nucleus of V; Vd, dorsal nucleus of V; Vv, ventral nucleus of V.

To characterize the ependymal cells in the killifish telencephalon, HCR targeting EPD was performed (Figure 4a), revealing the expected and exclusive presence of EPD‐RG4 in the tela choroidea, the thin layer surrounding the ventricular space (Figure 4b–e). To spatially map NE‐RG3, we visualized the expression of ZIC2, which was highly expressed in NE‐RG3 and to a lesser extent in Astro‐RG2 and EPD‐RG4 (Figure 4a). Of note, co‐labeling of ZIC2 and CX43 (expected in Astro‐RG2) was not detected (data not shown), ensuring the visualization of NE‐RG3 only with this marker in the telencephalon. ZIC2 expression was abundant ventrally, at the conjunction of the hemispheres and the posterior pallium, corresponding with neurogenic regions I and III, respectively (Figure 4e–g). To characterize the progenitor profile of NE‐RG3, we assessed co‐labeling of ZIC2 with SOX2 and PCNA (Figure 4a,h–q). All ZIC2^+^ cells in neurogenic regions I and III were positive for SOX2, indicative of the progenitor capacity of NE‐RG3, which was not restricted to the ventricular surface but also extended into the parenchyma. On the contrary, only a fraction of ZIC2^+^ cells were positive for the proliferation marker PCNA. The dividing NE‐RG3 cells were restricted to the ventricular border of neurogenic regions I and III. We could not observe ZIC2^+^ cells at the dorsal surface (Figure 4f,g).

### 2.7 NGPs are found spread throughout the neurogenic niches

**FIGURE 5:**
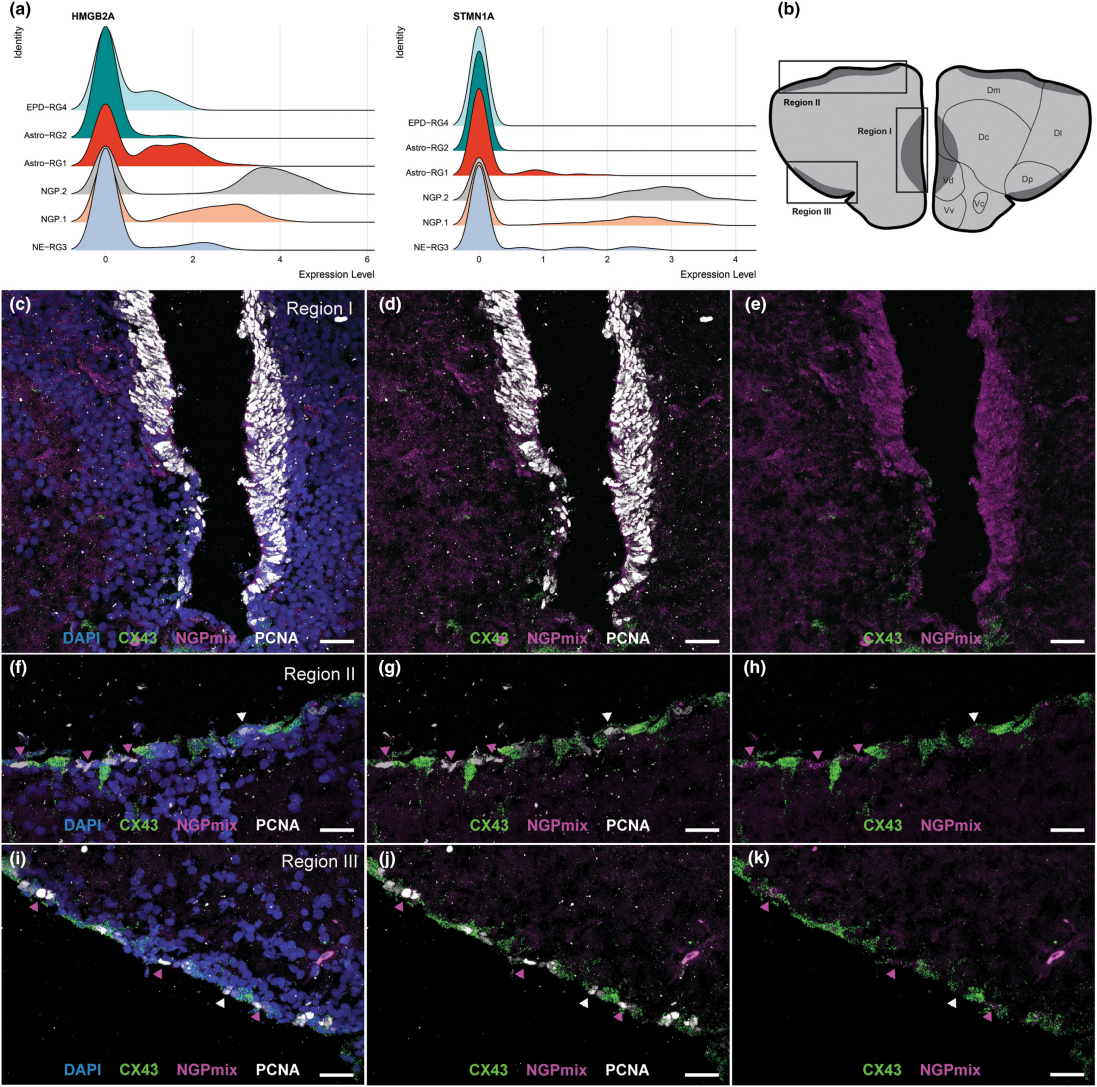
Complementary expression pattern of NGPs and NE‐RG3 present in the neurogenic niches. (a) Ridge plots show the density of cells expressing the deterministic markers for the NGPs: HMGB2A and STMN1A in six main progenitor cell types. (b) Schematic of the three neurogenic niches (I–III; dark grey) on a coronal section of the telencephalon. (c–k) Magnification of the three neurogenic regions from coronal sections labeled for CX43 (green), HMGB2A and STMN1A (= NGPmix, magenta), in combination with an immuno‐histochemical staining for PCNA (white) and a nuclear stain (DAPI, blue). In region I (c–e), a high abundance of highly proliferative NGPs (NGPmix^+^, PCNA^+^) can be observed, with a limited amount of CX43^+^ RGs at the ventral site of this zone. In region II (f–h) and region III (i–k), the NGPs are positioned in between Astro‐RG1 (CX43^+^) at the ventricular surface. Magenta arrowheads point to the NGPs (PCNA^+^, NGPmix^+^). White arrowheads point to a limited amount of PCNA^+^, NGPmix^−^, and CX43^−^ cells in between the NGPs and Astro‐RG1 (green), identified as a small subgroup of dividing RG3 (only PCNA^+^). Scale bar: 20 μm. Dc, central zone of D; Dl: lateral zone of D; Dm, medial zone of D; Dp, posterior zone of D; Vc, central nucleus of V; Vd, dorsal nucleus of V; Vv, ventral nucleus of V.

High expression of HMGB2A was found exclusively in the NGP.1/2 clusters, making it an excellent marker gene (Figure 5a). STMN1A, known to have an important role in adult neurogenesis, more specifically in the transition from precursor to postmitotic neurons, was also highly expressed in NGP.1/2 (Figure 5a) (Boekhoorn et al., 2014). Visualization of HMGB2A and STMN1A expression together (referred to as “NGPmix”) revealed the presence of proliferating NGPs (PCNA^+^ and NGPmix^+^) in all known telencephalic neurogenic niches (Figure 5b,c–k) (Tozzini et al., 2012). A clear difference in the abundance of NGPs could be observed between the neurogenic niches, with the medial region I having a denser cluster of NGPs (Figure 5c–e). On the contrary, at the dorsal and lateral ventricular surface, in regions II and III, a more scattered pattern was detected (Figure 5f–k). In these zones, the NGPs were dispersed in between Astro‐RG1 (CX43^+^) cells, which were mostly nondividing (PCNA^−^) (Figure 5f–k). The actively proliferating NGPs thus appeared dispersed in between less or nondividing RG populations in neurogenic regions, as well as grouped into a hotspot of proliferation at the subpallial neurogenic niche (region I) of the teleost telencephalon.

### 2.8 Aging changes cell type proportions and transcriptional signatures

**FIGURE 6:**
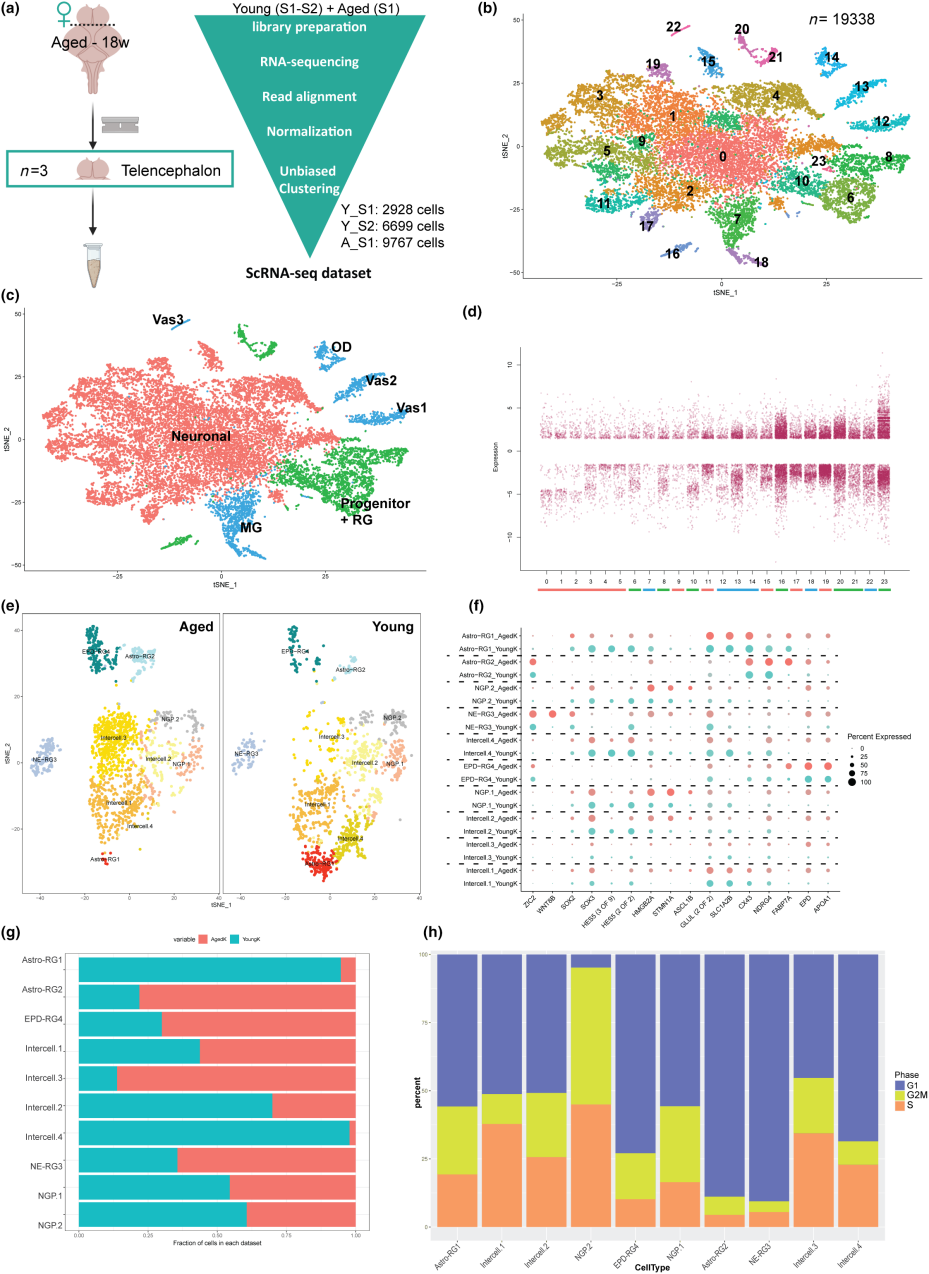
Impact of aging on telencephalic cell types. (a) Schema of single cell sequencing of young and aged telencephali. (b) TSNE Plot shows 23 identified cell clusters in merged young and aged dataset (Resolution 0.8). (c) TSNE plot shows the broad cell categories; color‐coded for Neuronal cells: pink, Progenitor cells: green, Other cells: blue. (d) Strip chart showing number of age‐dependent differentially expressed genes in the 23 individual cell types. Color coding below the x‐axis denotes the same cell types as in (c) (e) TSNE plot shows progenitor subtypes and their responses condition‐specifically. (f) Dot plot shows impact of aging on markers specific to progenitor subtypes. The size of the dot indicates the percentage of cells expressing the gene (0%–100%), the color indicates the expression level. (g) Altered progenitor subtype proportions upon aging. (h) Cell cycle phase proportion differences between aged progenitor subtypes indicating changes in their level of activity/quiescence.

We previously showed that aging dramatically hampers the capacity of the killifish telencephalon to regenerate neurons and repair after injury leading to a mammalian–like scar formation (Van houcke, Mariën, Zandecki, Vanhunsel et al., 2021). To understand the impact of aging on telencephalon cell types, particularly on the radial glia and proliferative progenitor subtypes identified in the young telencephalon, we profiled the transcriptome of individual cells in the aged telencephalon, at 18 weeks post‐hatching, and compared it to our young adult dataset of 6‐week‐old telencephalon (Figure 6a).

After quality control, data filtration and batch corrections (see Section 4), we obtained 9767 high‐quality cells (Table S1). To identify age‐dependent changes, the “young” and “aged” datasets were merged to a total of 19,338 high‐quality cells, and further unbiased clustering revealed 24 clusters (0–23, Figure 6b, Figure S3b–h). Using Seurat integration and label transfer methods, we found molecular anchors common between the two datasets. These represent pairwise correspondences between individual cells (one in each dataset), that we hypothesize originate from the same biological state. Further using distinct markers for 24 clusters in the merged dataset and specific markers for all cell types (Figure 1c), we could annotate all cells including similar non‐neural clusters, like microglia and oligodendrocytes, next to RG, progenitor, and neuronal clusters (Figure 6c, Table S5).

As expected, all clusters contained cells of young and old telencephalon, showing that none of the cell types was age‐specifically gained or lost (Figure S3a,b). Their transcriptomic profiles were also compared using discriminating markers for all cell types (Figure S4a). However, we noticed a major difference in clusters 0 (neuronal) and 7 and 18 (microglia), across the conditions, marked by a conspicuous rise in number of cells upon aging (Figure S3b). To understand the molecular impact of aging on the cell types, we performed pseudo‐bulk differential gene expression per cell cluster and condition (Figure 6d, Table S6). This analysis revealed that aging does not lead to many changes in gene expression in neuronal clusters, but progenitor clusters and other non‐neuronal cells appear more affected upon aging (Figure 6d). To elaborate on this finding and in line with our previous observations in young telencephalon, we focused our analysis on the progenitors.

To discover how aging affects the progenitor clusters (radial glia and proliferating cell types), we subclustered these cells (Figure 6e, Figure S4b). There were dramatic differences in the proportion of “young” versus “old” cells in specific clusters, while other clusters were much less affected (Figure 6f). In particular, Intercell.4 cluster as well as Astro‐RG1 contained proportionally much less “aged” cells, while Intercell.3, Astro‐RG2 and EPD‐RG4 had relatively more “aged” cells. Loss of the less mature Intercell.4 and gain of the mature Astro‐RG2 and EPD‐RG4 was expected to occur in the adult brain from our lineage prediction. The gain in Intercell.3 at the expense of Astro‐RG1 might point toward a shift in cell state, as the Intercell clusters represent transitional cell states. Compared to Astro‐RG1, Intercell.3 had downregulated expression of typical astroglia markers GLUL, SLC1A2B, and CX43 (Figure 6f). Comparing marker gene expression for progenitor clusters revealed almost no changes upon aging, which was expected, but we did observe a clear and specific increase in WNT8B expression in NE‐RG3 in the aged condition (Figure 6f, Table S7). Another aspect of progenitor aging we investigated was the impact on proliferation signature, as measured by expression of cell cycle‐related genes. Intriguingly, we found that NGP.1 became much less proliferative, with a larger proportion of cells with G2/M markers (Figure 6h). The latter seemed to be an overall trend appearing in many aged progenitor clusters. Another remarkable observation is the increased S‐phase markers in the intercell.1–3 clusters, suggesting these cell states might try to compensate for the loss of the proliferative capacity of the NGP, with relatively more cells in the S and G2/M phase compared to the young brain.

Besides changing proportions in cell states and types, we wondered whether cells within a cluster undergo transcriptional changes upon aging. Indeed, aging might not affect the cell type markers itself, but other gene programs underlying the cell type transcriptome. We assessed the overall impact of aging using a pseudo‐bulk approach and a differential expression analysis, for which we pooled the NGP populations to increase the power of the analysis. We compared the overall alterations induced due to aging by pseudo bulking young versus aging samples and this confirmed a clear inflammatory picture with genes such as APOEB, B2M, CCL5, CD68, etc. upregulated irrespective of cell types (Figure S4a,c, Table S8). We further performed KEGG pathway analysis of up‐ and down‐regulated genes per cell type (Figures S5–S7). For many clusters, we found similar pathways that were up‐or down‐regulated, such as oxidative phosphorylation, cellular senescence, P53 signaling, and pathways related to infection/inflammation which are known age‐dependent changes. We also observed downregulation of ribosomal genes in neuroepithelial, non‐glial, and ependymal subtypes as well as intercell states. In several cases we found the same pathways affected in both directions, suggesting deregulation of different genes in the same pathway. In addition, we found pathways that changed in a very cell‐type specific manner (Table S9). Intercell.3, the cluster that becomes more prominent upon aging, had a clear signature of genomic reorganization. The highest number of enriched pathways was discovered to be downregulated in aged NE‐RG3 and NGP. In the case of NGP, *MAPK pathway*, *Cell cycle*, *FoxO*, and *Notch signaling* were downregulated, in line with the reduced neurogenic potential of aged NGP cells (Table S10a). Notably, the pathways downregulated in the NE‐RG3 were *mTOR*, *NOD‐like receptor*, *tight and adherens junction*, and *Wnt signaling* (Table S10b). In NE‐RG3, we also noticed a bulk of important pathways upregulated (*Wnt signaling*, *senescence*, *phagosome*, *and NOD‐like receptor*) confirming that major changes occur in the cell type upon aging (Figure S5b,d).

Taken together, our data show that aging induces profound inflammation‐related transcriptional changes, but also cell‐type specific changes that fit with the observed decline in neurogenic activity from the progenitors, as well as novel pathways that are affected in a cell‐type and age‐dependent manner.

## 3 DISCUSSION

The short‐lived turquoise killifish *N. furzeri* has become a validated model for age‐related disease research over the last years (de Bakker & Valenzano, 2023; Ruparelia et al., 2024; Van houcke, Mariën, Zandecki, Seuntjens et al., 2021; Van houcke, Mariën, Zandecki, Vanhunsel et al., 2021). Our previous research has shown that unlike other teleosts, the aged killifish telencephalon is not capable of seamless neuronal repair, similar to mammalian brain (Van houcke, Marië, Zandecki, Vanhunsel et al., 2021). To better understand the cellular basis of age‐related cognitive decline, profound knowledge of the cell types present in the brain is imperative. Here, we performed single‐cell RNA sequencing of female young and aged telencephalon of the GRZ strain, which is the shortest‐lived laboratory killifish strain. We discovered diversity in neuronal and non‐neuronal cell types, the variety of progenitor cell types driving neuro‐and gliogenesis and the impact of aging on the proportion and transcriptional signature of these cells. Our extensive dataset will be a valuable tool to further mine the cellular impact of aging on the whole telencephalon.

The adult killifish telencephalon cell types resemble cell types found in other vertebrate telencephalon single‐cell studies. We annotated common vertebrate cell types such as neurons, radial glia, oligodendrocytes, microglia, and vasculature‐associated cells. We also found excitatory and inhibitory neuronal lineages, as noticed in other vertebrates and teleostean models. In our study, we noticed a differential expression of *EOMESA* in distinct excitatory lineages reflecting on regional subtypes. There is similar evidence in zebrafish telencephalic niches, wherein *EOMESA* expression is stronger in the lateral than medial pallium (Ganz et al., 2015). We probed the diversity of neuron subtypes within the telencephalon using a subclustering approach and indeed found diverse sets of excitatory and inhibitory (inter)neuron cell profiles, revealing that our dataset lays the foundation for exciting future studies exploring the neural diversity in this teleost model.

As a teleost, the killifish has an everted telencephalon, with progenitor cells in neurogenic niches located at the ventricular surface. These niches were populated by a mixture of quiescent Astro‐RG and highly proliferative NGP cells, a stem cell type found to be present already in the 2‐week‐old killifish telencephalon (Coolen et al., 2020). We found two subtypes of Astro‐RG located in distinct regions of the telencephalon; Astro‐RG1 seems to be the predominant type that is spread over the dorsal pallium surface, while Astro‐RG2 is confined to the subpallial medial neurogenic niche. A similar subdivision in radial glia subtypes has been described (Adolf et al., 2006; Grandel et al., 2006), and because of their position in the medial subpallium, Astro‐RG2 might be quiescent progenitors linked to neurogenesis of olfactory lobe neurons. Other (single‐cell) profiling of zebrafish radial glia has been performed on sorted Her4.1^+^ progenitors, but killifish Astro‐RG2 do not express HES5, the Her4 orthologue, and so the extent of astroglia diversity might have been overlooked in these studies (Cosacak et al., 2019; Lange et al., 2020).

The ZIC2^+^ NE‐RG3 population was spatially overlapping with a described group of neuroepithelial cells at the edge of the lateral pallium in zebrafish, suggested as the source of radial glia cells in adult life (Labusch et al., 2020). Other studies in zebrafish have revealed that the marker ZIC2a along with SIX3 is known to negatively regulate Hedgehog‐dependent transcription in the developing forebrain (Sanek et al., 2009). The killifish NE‐RG3 cells seemed primarily quiescent, and our trajectory analysis indicated a role for these cells as a source to give the brain the possibility to respond swiftly to injury or disease, besides supporting explosive brain growth in early postnatal life (Coolen et al., 2020).

As found in the larval and adult telencephalon, NGPs comprise the most actively proliferating cell group in both pallium and subpallium. Sub‐clustering indicated two prevalent cell states NGP.1 and NGP.2 linked to different phases in the cell cycle (S and G2/M, respectively) rather than different spatial organization. We hypothesize that cycling NGPs might swap groups depending on their cell cycle phase. The decision of cells to become glial or neuronal cells is potentially made within these fast‐proliferating NGPs as they are the common progenitor to both glial cell types and neurons. In the developing killifish telencephalon, we recently indeed found evidence for a subtype of NGPs that seems to be committed to the (glutamatergic) neuronal lineage (Zandecki et al., 2024). Since RGs do not seem to support neurogenesis as much as NGPs in the killifish telencephalon (Coolen et al., 2020; Van houcke, Mariën, Zandecki, Vanhunsel et al., 2021), they are primarily contributing to glial functions, including astrocytic and ependymal roles. In the zebrafish brain, diverse motile ciliated and non‐ciliated cell types within ependymal lineages have been discovered. We found EPD‐RG4 cells that are devoid of typical ciliary component‐associated genes, hence further analyses are needed to explore the inherent diversity within the killifish ependyma (Jurisch‐Yaksi et al., 2020).

Aging had an undeniable impact on cell transcriptomes. Some of our findings corroborate what has been found in the mouse telencephalon upon aging: it has an aggregated impact on the transcriptome, with signs of increased inflammatory responses, but also particular cell type‐specific aging signatures (Ximerakis et al., 2019). As the killifish telencephalon is much less adept to repair when aged (Van houcke, Mariën, Zandecki, Vanhunsel et al., 2021), we sought to retrieve molecular signatures of progenitor‐related aging. Upon aging, the loss of Astro‐RG1 cell numbers was accompanied by an increase in the intermediate states (Intercell.1, 3) and an overall increase in the number of cells with a G2/M profile. As the number of mitotic cells drastically reduces upon aging in the killifish telencephalon (Van houcke, Mariën, Zandecki, Vanhunsel et al., 2021), this suggests a profile of replicatively senescent cells that are arrested in G1/G2 without being able to progress to cell division (Mao et al., 2012). Quiescence linked to G2 has been observed in Drosophila, where larval neuroblasts can be rapidly induced to divide by insulin signaling (Otsuki & Brand, 2018). G2‐arrested progenitors have not yet been observed in vertebrates, and more validation is needed to prove their appearance upon aging.

Aging impacted the gene expression profiles to a different extent in specific cell types. NE‐RG3 can be categorized as the most perturbed cell type as it contained the highest number of differentially expressed genes. In line with the reduced proliferative and neurogenic capacity of aged NGPs, we found “cell cycle” and “Notch signaling” to be linked to a downregulated gene list. The increase of genome organization‐related processes in aged Intercell.3 cells is reminiscent of the massive change in chromatin organization that accompanies senescence (Shaban & Gasser, 2023). We thus hypothesize that upon aging, Astro‐RG1 do not disappear but downregulate marker expression, transform to an “intercell‐type” profile and become senescent. In a previous study, we indeed showed increased senescence‐associated B galactosidase labeling of aged ventricular progenitors, including some with RG morphology, confirming a senescent cell state (Van houcke et al., 2023). Another interesting observation is around ribosomal transcript changes in cell types. In aging mice, an upregulation of ribosomal genes in neurons, but downregulation in progenitors has been observed (Frenk & Houseley, 2018). Our data corroborate this finding with several progenitor cell types showing downregulation of ribosomal genes.

Important and evolutionarily conserved metabolic pathways such as mTOR and Wnt in NE‐RG3 and MAPK, FOXO, and Notch in NGP were found to be dysregulated (Figure S5c,d). FOXO, WNT, and mTOR are involved in nutrient sensing, which link nutrient availability with other cell functions that play important roles in the maintenance of stem cell quiescence during aging (Yun, 2015). Our bulk proteomics study of the aging killifish telencephalon also showed comparable changes in mTOR, Wnt, and MAPK pathways which is directly linked to cellular senescence and apoptosis (Van houcke et al., 2023). Characterizing molecular targets and/or inhibiting these pathways will be vital in developing future therapeutic strategies to reinstate neuroregeneration in the brain of aged killifish. Having a clear view of cell type diversity, relationships and spatial organization is an important first step in understanding the age‐dependence of the neurogenic potential of this emerging animal model. We note that despite the discovery of the major populations of cells in the adult telencephalon, there is certainly additional diversity to be explored in future studies. With this study, we set the stage for future work to address how age‐related injuries and diseases influence neurogenic and gliogenic landscapes.

## 4 METHODS

### 4.1 Fish strains

Given the sex‐dependent variation in aging rates, all experiments were performed exclusively on adult female 6‐week‐ and 18‐week‐old African turquoise killifish (*N. furzeri*), from the inbred GRZ‐AD strain. One male was housed with three females in 3.5 L tanks, containing a nontransparent T‐tube for hiding, in a multilinking Tecniplast ZebTec aquarium system under standardized conditions; temperature 28°C, pH 7, conductivity 600 μs, 12 h/12 h light/dark cycle. From hatching, the fish are fed twice a day with Artemia salina (Ocean Nutrition), 2 weeks after hatching, mosquito larvae (*Chironomidae*) are added to their diet. From 5 weeks onwards, the fish are only fed with mosquito larvae. All experiments were approved by the KU Leuven ethical committee in accordance with the European Communities Council Directive of 20 October 2010 (2010/63/EU).

### 4.2 Tissue collection and processing for spatial analysis of gene expression

Fish were euthanized in 0.1% buffered tricaine (MS‐222, Sigma Aldrich) and perfused with phosphate‐buffered saline (PBS) and 4% paraformaldehyde (PFA, 8.18715, Sigma‐Aldrich, in PBS) (Mariën et al., 2022; Van houcke, Mariën, Zandecki, Vanhunsel et al., 2021). Brains were carefully dissected and fixed overnight at 4°C in 4% PFA. Brains were washed three times in PBS and transferred overnight to 30% sucrose in PBS at 4°C. Next, brains were embedded in 30% sucrose, 1.25% agarose in PBS. Using a CM3050s cryostat (Leica), 10 μm‐thick coronal sections were cut and collected on SuperFrost Plus Adhesion slides (10149870, Thermo Fisher Scientific). The cryostat temperature was set to −28°C for the chamber and −26°C for the object. Sections were stored at −20°C until the start of the HCR.

### 4.3 Hybridization chain reaction

The probe pair pools targeting CX43, SLC1A2, EPD, ZIC2, STMN1A, and HMGB2A (Table S11) were generated and validated as described in detail before (Elagoz et al., 2022; Van houcke, Mariën, Zandecki, Vanhunsel et al., 2021). The selected probe pairs were ordered via Integrated DNA Technologies, Inc (IDT). The HCR protocol (HCR v3.0 (Choi et al., 2018)) is based on the protocol of Choi et al. (2018), adapted for cryosections as described in Van houcke, Mariën, Zandecki, Vanhunsel et al. (2021). In case HCR was combined with immunohistochemistry (IHC), the Proteinase K permeabilization step was removed and the rehydration steps were as follows: the sections were washed in PBS‐DEPC three times and once in 0.3% Triton‐X‐100 in PBS. Later, they were washed again in PBS, followed by a washing step in 5× SSCT (0.1% Tween‐20 in saline‐sodium citrate buffer (SSC)). After the hybridization and amplification steps, the slides were washed three times in 5× SSCT before proceeding with the IHC protocol.

### 4.4 Immunostaining after hybridization chain reaction

After HCR, the sections were washed in PBST (0.1% Tween‐20 in PBS) before blocking with 20% normal goat serum (S26, Sigma‐Aldrich) or normal donkey serum (D9663, Sigma‐Aldrich), depending on the secondary antibody, in Tris‐NaCl blocking buffer (TNB) for 2 h at room temperature (RT). After blocking, the sections were incubated with the primary antibody diluted in Pierce Immunostain Enhancer (Thermo Fisher Scientific) (Table S12). After an incubation of 24 h, the sections were washed with PBST and incubated with the secondary antibody in TNB for 2 h. Finally, the sections were rinsed with PBS followed by nuclear staining with 4′,6‐diamidino‐2‐fenylindool (DAPI, 1:1000 in PBS, Thermo Fisher Scientific) for 30 min. Afterward, the sections were mounted with Mowiol (Sigma‐Aldrich).

### 4.5 Imaging

Microscopic images for Figure 3 were acquired and processed using a Leica DM6 upright microscope (20×) and LAS X software (Leica Microsystems). Channels were intensified and merged using Fiji (Schindelin et al., 2012). Overview (20×) and high magnification (63×) images for Figure 4 were obtained using confocal microscopy (LSM 900 with Airyscan 2, ZEISS). Basic processing (orthogonal projections and intensity adjustment) was performed using ZEN software (ZEISS ZEN lite 3.7) To obtain the high magnification (63×) images for Figure 5, confocal microscopy was used (Fluoview FV1000, Olympus). The images were further processed (orthogonal projections and intensity adjustment) with Fiji (Schindelin et al., 2012).

### 4.6 Single‐cell suspension

Fish were euthanized in 0.1% buffered tricaine (MS‐222, Sigma Aldrich). The blood was removed by intracardiac perfusion with cold, sterilized PBS (Mariën, et al., 2022; Van houcke, Mariën, Zandecki, Vanhunsel et al., 2021). Next, telencephali were extracted and immediately placed into cold DMEM/F12 (Life Tech, Invitrogen). Telencephali were transferred into sterilized, freshly prepared papain solution (250 μL papain (P3125, Sigma Aldrich), 100 μL 1% DNase I (DN25, Sigma Aldrich®), 200 μL L‐Cysteine (12 mg/ mL, Sigma Aldrich), in 5 mL DMEM/F12 (Life Tech, Invitrogen)) and digested for 10 min at 37°C. Hereafter, telencephali were dissociated by gently triturating with a cut pipet tip and placed back for 10 min at 37°C. This procedure was repeated until the brain tissue was completely dissociated into single cells. The suspension was then put through a polypropylene strainer (35 μm, Falcon) and 2 mL of ice‐cold, sterilized, freshly prepared washing solution 650 μL D‐(+)‐glucose 45% (G8769, Sigma Aldrich®), 500 μL HEPES (1 M, Thermo Fisher), 5 mL FBS (Life Tech, Invitrogen) in 1X DPBS (Life Tech, Invitrogen) was added. Cells were centrifuged for 10 min at 500*g* at 4°C and the supernatant was discarded. Next, debris was removed via the debris removal solution protocol (130‐109‐398, Miltenyi Biotec) to ensure high viability of the suspension. Finally, cells were resuspended in cold sterilized 0.04% BSA in PBS. For each sample, three telencephali were pooled. Cell viability was measured at the KU Leuven Genomic Core. A viability of 97.4% was retained after the whole procedure. A detailed description of the single‐cell suspension protocol is described here (Mariën, Arckens et al., 2024). Young female sample S1 and old female sample S1 (Figures 1a and 6a) have been run in parallel from sample preparation to data acquisition. To compensate for the lower number of cells in the young sample post scRNA sequencing, most probably related to the smaller size of young animals/brains, we supplemented this sample with an extra sequenced replicate (young sample S2).

### 4.7 Telencephalon SMRT‐sequencing

The Iso‐Seq method produces full‐length transcripts using Single Molecule, Real‐Time (SMRT) Sequencing, thus attaining high accuracy with better genome coverage (Eid et al., 2009; Wang et al., 2016). We pooled 2–3 telencephali (6‐week‐old) and extracted whole RNA using the RNAEasy Micro kit (Qiagen), with blood. Final RNA quality and integrity were assayed using the DNA 12000 kit on Bioanalyzer (Agilent). Further, this was subjected to PACBIO SMRT Sequencing as recommended in Pacbio protocol for Sequel systems. The SMRT sequencing was performed on the PacBio Sequel at the Genomics Core at KU Leuven (Belgium) using the Clontech SMARTer PCR cDNA Synthesis Kit (recommended by Pacbio). Multiple parallel PCR reactions were set after the first strand synthesis employing the PrimeSTAR GXL DNA Polymerase kit. Next, cDNA of a specific length was selected by splitting these PCR reaction products into two fractions, purifying one with 1X AMPure PB Beads and the second with 0.4X AMPure PB Beads, and subsequently mixing both purified products in an equimolar fashion. These libraries were constructed using the SMRTbell template prep kit 1.0. They were sequenced employing Sequel Sequencing, and Binding kits 3.0 on a Sequel I platform with SMRTCells V3.0 LR, allowing a movie collection time of 20 h. The mRNAs were selected by poly‐A tails, without 5'cap selection, which was taken into account in the subsequent analysis. The reference genome, gene annotation, and GO terms were downloaded from the *N. furzeri* Information Network Genome Browser (NFINgb). We further used the recommended Pacbio ISOSEQ pipeline to analyze the sequencing data with in‐house modifications. We further used the GFFCompare tool to create an updated killifish‐specific annotation file by merging the *N. furzeri* reference and Iso‐Seq derived full length transcriptome from 6‐week‐old telencephalon and its specific annotations (Ayana et al., 2024). This helped us improve our final genomic annotation file and ensured higher mapping accuracy and coverage for performing Seurat‐based single‐cell sequencing analyses.

### 4.8 Telencephalon 10X genomics sequencing

10X Genomics is known to outperform other droplet‐based sequencing methods in terms of bead quality and barcode detection efficiency. To prepare the samples for single‐cell sequencing using 10X genomics, all cells isolated from three female killifish telencephali were pooled per sample (6‐week‐old, two samples, 18‐week‐old, one sample; Figures 1a and 6a). Subsequently, the single‐cell suspension was carefully mixed with a reverse transcription mix before loading the cells on the 10X Genomics Chromium system. Library preparation was done using 10X Genomics droplet‐based sequencing with the 10X Chromium Single Cell 3′ (v3 Chemistry) reagents kit. The cells were lysed within the droplet and they released polyadenylated RNA bound to the barcoded bead, which was encapsulated with the cell. Following the guidelines of the 10X Genomics user manual, the droplets were directly subjected to reverse transcription, the emulsion was broken, and cDNA was purified using Silane beads. After the amplification of cDNA with 10 cycles, purification and quantification was performed. The 10X Genomics single‐cell RNA‐sequencing library preparation involving fragmentation, dA‐tailing, adapter ligation, and 12‐cycle indexing PCR was performed. After quantification, the libraries were sequenced on an Illumina NovaSeq machine using a HighOutput flowcell in paired‐end mode (R1: 26 bp; I7: 8 bp; R2: 50 bp), thus generating 100 million fragments.

### 4.9 Quality check of single‐cell sequencing data

Quality testing of the sparse data matrix (containing genes and cell type sequences) yielded 9627 cells. (Table S1). Sequencing metrics showed the presence of 25,800 mean reads per cell with an average gene per cell ratio and absolute transcript or unique molecular identifier (UMI) per cell ratio of 828 and 1687, respectively (Table S1). Overall, the cells utilized were sequenced at a depth to obtain optimum coverage of the *N. furzeri* genome (87%). The sequenced reads also confidently mapped to 69% of the killifish transcriptome. On average we detected 17,713 unique genes in both samples (9616 cells). Overall, we could capture high‐quality reads (~97.2% valid cell barcodes) and attain average 57.5% sequencing saturation (Table S1). In case of aged data, after QC, and data filtration 9767 cells were obtained. Further details of ISOSEQ transcriptome mapping for aged data is available (Table S1).

### 4.10 Single‐cell sequencing data analysis

Raw data were processed with the Cell Ranger 3.0 software. First, we built the reference for CellRanger using the “mkgtf” command (default parameters). The killifish genome (GRZ Assembly, 05/2015 (Valenzano et al., 2015)) as well as the latest NCBI gene annotation (NFINgb), in conjugation with the in‐house sequenced killifish ISOSEQ annotation files were used to specify the reference in the “mkgtf” command. Specifically, the two GTF files from PACBIO long‐reads specific to telencephalon and the NFIN database reference transcripts were merged using the GffCompare tool. This was followed by the “count” command as part of CellRanger, the option of “– expect‐cells” was set to 2500 (all other default options). The results included the feature barcode matrices usable for downstream analysis in R.

### 4.11 Data analysis with Seurat

All matrices from CellRanger 3.0 were read by the Read10X function using Seurat 4.9 package (Hao et al., 2024; Stuart et al., 2019). Initially, the two samples were processed separately before integration. Initial quality check included removal of ribosomal genes (RPS/L genes) and filtration/removal of cells using the following criteria: (a) cells having >5% mitochondrial content, (b) cells <500 and >2500 unique genes. We further removed genes/features detected in at least two cells (min.cells parameter) and filtered the top 2000 highly variable genes for further analysis. The remaining variable cells and genes were used for downstream analysis. Further, we scaled, normalized, and checked the variability of the data using the single SCTranform function. Initially, two Seurat SCTransformed objects, adult killifish and zebrafish scSequencing data as a reference (Cosacak et al., 2019) were used to generate a combined object with IntegrateData function for marker gene identification studies. Post optimization, we only used two killifish samples as one integrated object (objects) using FindAnchors and IntegrateData functions and further performed progenitor‐based sub‐clustering. We performed unbiased clustering on the top 30 Principal components which has been represented in the form of dimensionality reductive T‐distributed Stochastic Neighbor Embedding (TSNE) at optimized perplexity = n/100^73^, and Uniform Manifold Approximation and Projection (UMAP) algorithms. Progenitor sub‐clustering was performed by sub‐setting pertinent clusters from “all cells” object and rerunning the Seurat analysis. Similar downstream analyses were performed for the sub‐setted progenitor dataset; optimized resolution = 0.4, 20 PCs.

### 4.12 Cell type identification

Cell type identification‐related plots (Feature plots and dot plots) were generated by Seurat 4.9, and cell types were determined by the expression of tested marker genes in the laboratory that define specific cell types and known expression in related species, zebrafish, and humans. We also removed a population of cells occurring due to possible contamination (van den Brink et al., 2017). The marker genes for all resultant cell populations (All cells, PCs, and NCs) were calculated by using FindAllMarkers and FindMarkers function with options min.pct = 0.25, thresh. use = 0.25 and test.use = MAST (Mast V1.24). Using the FindMarkers and FindAllMarkers function, we ascertained the ranking of all differentially expressed marker genes (threshold: PCT ≥0.25) per cell cluster, with associated significance value (corrected *p*‐value ≤0.05). The same parameters were applied for the sub‐clustering analyses.

### 4.13 Lineage inference analysis with Slingshot

To elucidate the cell lineage on pseudotime, we made a subset of clustered PC cells (n_cells = 1303), and further converted that Seurat object to a Slingshot object via Slingshot 2.6 (Street et al., 2018). Primarily, this is done based on the differential gene expression between cell types by constructing a minimum spanning tree (MST) on cells in a reduced‐dimensionality space created by independent component analysis (ICA), and orders cells via a PQ tree along the longest path through this tree. The subset of all cells included NGP.1/2, Astro‐RG1/2, NE‐RG3, and EPD‐RG4 in case of PCs. The “start. cluster” was set to “NE‐RG3” and multiple lineages in “curves” mode were analyzed. The estimated size factor was set to 0.6 as the data were already normalized by Seurat. The cells were colored according to the colors on the Seurat TSNE plots. We further performed NB‐GAM (negative binomial generalized additive model (NB‐GAM)) analysis using tradeSeq 1.2, an R package that allows analysis of gene expression along trajectories. We used the patternTest function, which implements a statistical method to check whether the smoothed gene expression is equal along pseudotime between two or multiple lineages. We also checked for early drivers of differentiation using the specifying the knots or branch points of interest, and logging in the gene‐specific count information using the plotGeneCount and plotSmoothers function.

### 4.14 Aging data integration

The aged killifish sample was preprocessed and filtered similarly to young samples. Using SCTransform, we scaled and normalized the object before integrating it into the young dataset. Further, via Seurat's internal integration method (CCA) we searched for integration anchors across the samples, ensuring accurate label transferring. Using IntegrateData function, we performed a batch‐corrected integration between samples and this object was further used for all integrated analyses. Using PCA, we found the optimized number of PCs for further dimensionality reduction testing. We performed unbiased clustering on the top 40 Principal components which has been represented in the form of dimensionality reductive T‐distributed Stochastic Neighbor Embedding (TSNE) at optimized perplexity depending on the number of cells. The optimized resolution was 0.8. We further used the RNA assay to perform normalization for finding conserved cell‐type specific markers using FindAllMarkers (test.use = MAST 1.2, only.pos = F, min.pct = 0.25).

We further took a pseudo‐bulking approach and aggregated expression between conditions (young vs. aged) to analyze overall impact due to aging (Wilcox statistical test); log_2_FC <>1.2. We also performed pseudo‐bulking across identified cell types and divided all cell types as per condition (celltypeX_young vs. celltypeX_aged) to perform differential expression analyses (Wilcox statistical test; log_2_FC > ±1.2). The resultant gene lists for each cell type were used for KEGG pathway analyses using ShinyGO 0.77 (Ge et al., 2020) (Figures S5–S7).

### 4.15 Cell cycle scoring for cell types

To examine the cell cycle variations in differentiating Progenitor subtypes, we assigned a score using S and G2M phase genes present in each cell. This was done using the CellCycleScoring function in Seurat 4.9 and the cells with low S and G2M scores were assigned a G1 score. This way, we could ascertain the changes in cell types and/or states within the whole cell population (only young PCs) and upon aging (only aged PCs).

## AUTHOR CONTRIBUTIONS

R.A. was involved in conceptualization, experimental design, bioinformatics analysis, application development, data visualization, writing: draft, review and editing. C.Z. was involved in experimental design, HCR experiments and analysis, data visualization, writing: draft, review and editing. J.V.H. and V.M. were involved in single‐cell suspension preparation, writing: review and editing. L.A and E.S. were involved in study supervision, conceptualization, experimental design, writing: review and editing. All authors approved the submission.

## FUNDING INFORMATION

This work was supported by the Fonds voor Wetenschappelijk Onderzoek (FWO Vlaanderen): research grant numbers: G0C2618N, G0C9922N; research infrastructure (former Hercules) funding: I013018N; and personal fellowships to JVH (FWO‐1S00318N; KU L‐VTI‐23‐00197); KU Leuven equipment and research grants (KA/16/020; KA/20/013; C3/21/012).

## CONFLICT OF INTEREST STATEMENT

The authors declare no competing interests.

## Supporting information


Figures S1–S7.

Tables S1–S12.

## Data Availability

All 10X sequencing data have been deposited in GEO (http://www.ncbi.nlm.nih.gov/geo/) under BioProject ID PRJNA718608 under sample IDs: young (SRR14110665, SRR24058857) and aged (SRR27989201). All code generated has been deposited on our Github page (https://github.com/ayanarajagopal/YA‐Killifish_scSeq).
