# Supplementary material for: Single‐cell sequencing unveils the impact of aging on the progenitor cell diversity in the telencephalon of the female killifish N. furzeri
Source: Aging Cell. 2024 Jul 1;23(10):e14251. doi: 10.1111/acel.14251 (PMC11464125; doi:10.1111/acel.14251)
Supplement: Supplementary file 1 — Figures S1–S7. [file ACEL-23-e14251-s002.zip › FiguresS1-S7.docx]

Supplementary Figure 1. Quality metrics of initial clustering. (a) Sample-wise overlap of main cell clusters identified in 1D. (b) TSNE plot shows 23 cell types and their clustering between samples. (c) Heatmap of the top 10 markers (ordered by p-value) for the main cell clusters described in 1b. (d) Stacked violin plot for number of genes per cell (nGene) and number of reads per cell (nUMI) per cell type. (e) Violin plots for the number of genes per cell (nGene), number of reads per cell (nUMI), mitochondrial gene percentages (percent.mt) and ribosomal gene percentages (percent.ribo) per cell. (f) Elbow plot shows the most variable principal components among all cells. (g) Scatter plot showing no. of genes/transcripts detected per cell confirms the high quality of the dataset. (h) Scatter plot and correlation analysis between nUMI and nGene in all cells with sample specificity.

Supplementary Figure 2. Progenitor subtype identification and categorization. (a) Strategy for sorting progenitors for sub-clustering (PCNA, GLUL++); density plot shows combined percent cells expressing PCNA and GLUL. (b) Discriminating markers of 2 astroglia subtypes. (c) Violin plot shows the expression of genes associated with HES-ID developmental patterning that govern the cell state gene signatures that promote neurogenesis. Fold changes per gene are mentioned on the y-axis. (d) i) Feature plot reveals top differential genes across lineages (scale: yellow to red). (ii) Pseudotime-based plot shows the expression changes across individual lineages in top differential genes.

Supplementary Figure 3. Quality metrics of initial clustering. (a) TSNE plot shows composition of young and aged killifish datasets; sample-wise overlap revealing minimal batch effects. (b) TSNE plot shows 24 cell types and their clustering between conditions. (c) Heatmap of the top 10 markers (ordered by p-value) for the main cell clusters described in 6b. (d) Stacked violin plot for number of genes per cell (nGene) and number of reads per cell (nUMI) per cell type irrespective of condition. (e) Violin plots for the number of genes per cell (nGene), number of reads per cell (nUMI), mitochondrial gene percentages (percent.mt) and ribosomal gene percentages (percent.ribo) per cell. (f) Elbow plot shows the most variable principal components among all cells in merged dataset. (g) Scatter plot showing no. of genes/transcripts detected per cell confirms the high quality of the merged dataset. (h) Scatter plot and correlation analysis between nUMI and nGene in all cells with condition specificity.

Supplementary Figure 4. (a) All differentially expressed genes during aging irrespective of cell type information. Highlighted genes include inflammatory/immune response genes on the right, and cell cycle and replication associated genes on the left of the plot. (b) Cell proportions in young and aged telencephali; colors in accordance with TSNE plots (2a, 6e). (c) Full list of gene markers for all comparable cell types in merged dataset (Suppl. table 5); Young (green), Aged (purple).

Supplementary Figure 5: Differential expression across root progenitor cell types during aging. (a-b) KEGG pathways significantly dysregulated in the down- and upregulated gene list of NGP. (c-d) KEGG pathways significantly dysregulated in the down- and upregulated gene list of NE-RG3. Note: A higher fold enrichment score (number of genes/pathway size) indicates higher significance.

Supplementary Figure 6: Differential expression across astroglia and ependymoglia cell types during aging. (a-b) KEGG pathways dysregulated in the down- and upregulated gene lists of astroglia type Astro-RG1. (c-d) KEGG pathways dysregulated in the down- and upregulated gene lists of Astro-RG2. KEGG pathways dysregulated in the down- and upregulated gene lists of astroglia type EPD-RG4.

Supplementary Figure 7. Differential expression across transition cell states or intercell sub-types during aging. (a-b) KEGG pathways dysregulated in the down- and upregulated gene lists of Intercell.1. (c-d) KEGG pathways dysregulated in the down- and upregulated gene lists of Intercell.2. (e-f) KEGG pathways dysregulated in the down- and upregulated gene lists of Intercell.3. (g) KEGG pathways dysregulated in the upregulated gene lists of Intercell.4. Note: No pathways were dysregulated in the downregulated gene list of Intercell.4.
