# Supplementary figures and images for: Single‐cell sequencing unveils the impact of aging on the progenitor cell diversity in the telencephalon of the female killifish N. furzeri
Source: Aging Cell. 2024 Jul 1;23(10):e14251. doi: 10.1111/acel.14251 (PMC11464125; doi:10.1111/acel.14251)

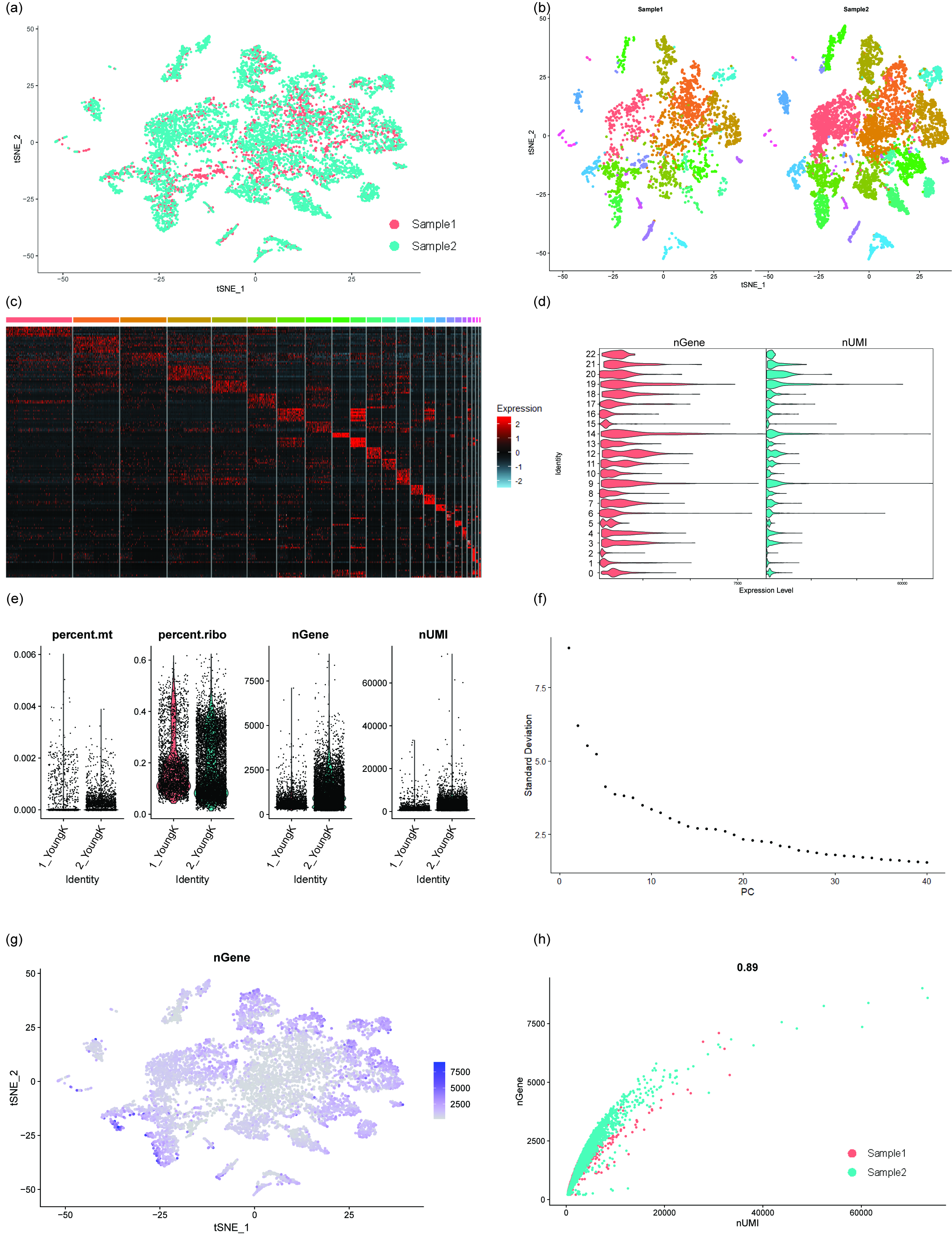

Supplement: Supplementary file 1 — Figures S1–S7. [file ACEL-23-e14251-s002.zip › acel14251-sup-0001-FigureS1.tif]

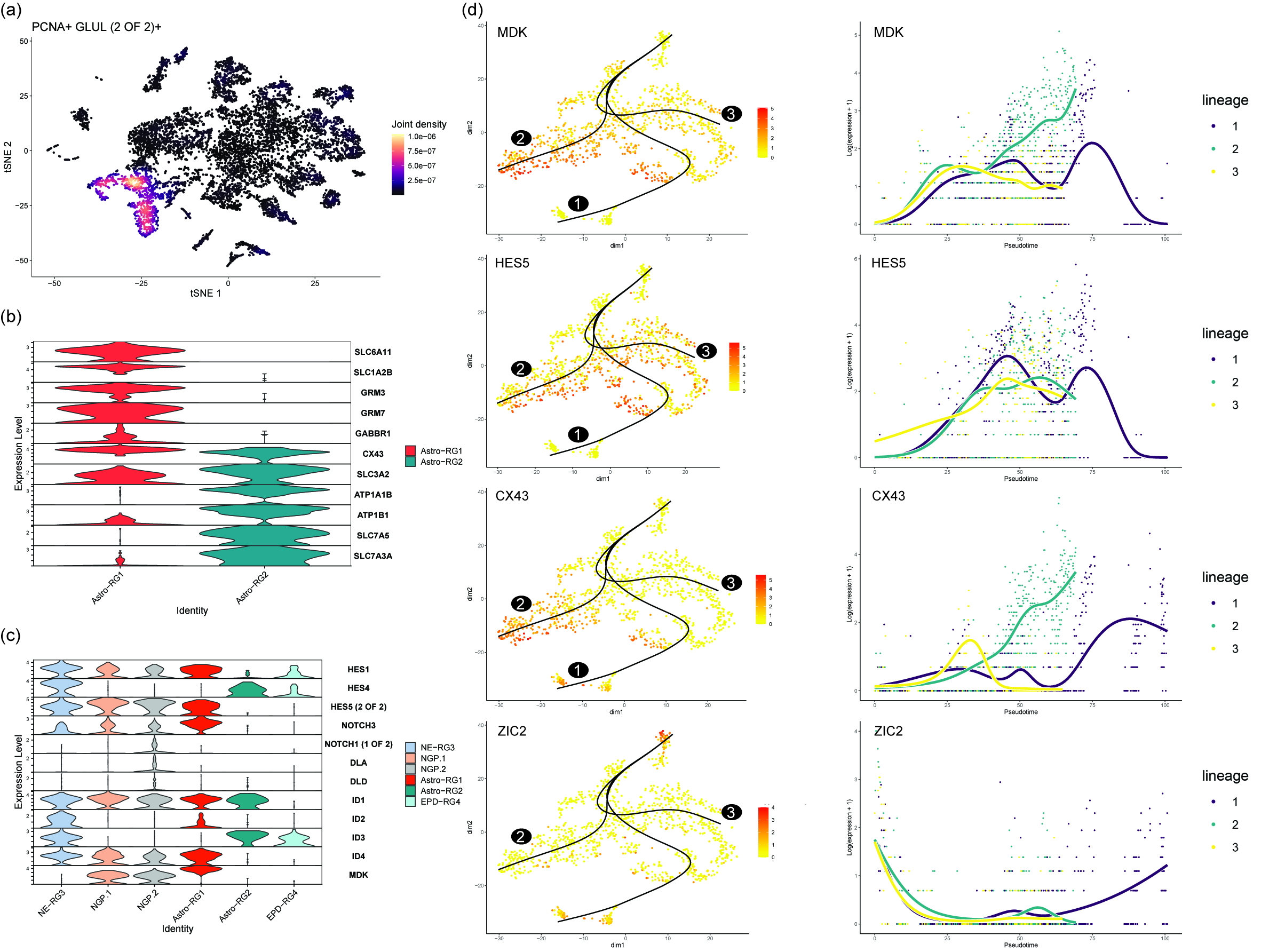

Supplement: Supplementary file 1 — Figures S1–S7. [file ACEL-23-e14251-s002.zip › acel14251-sup-0002-FigureS2.tif]

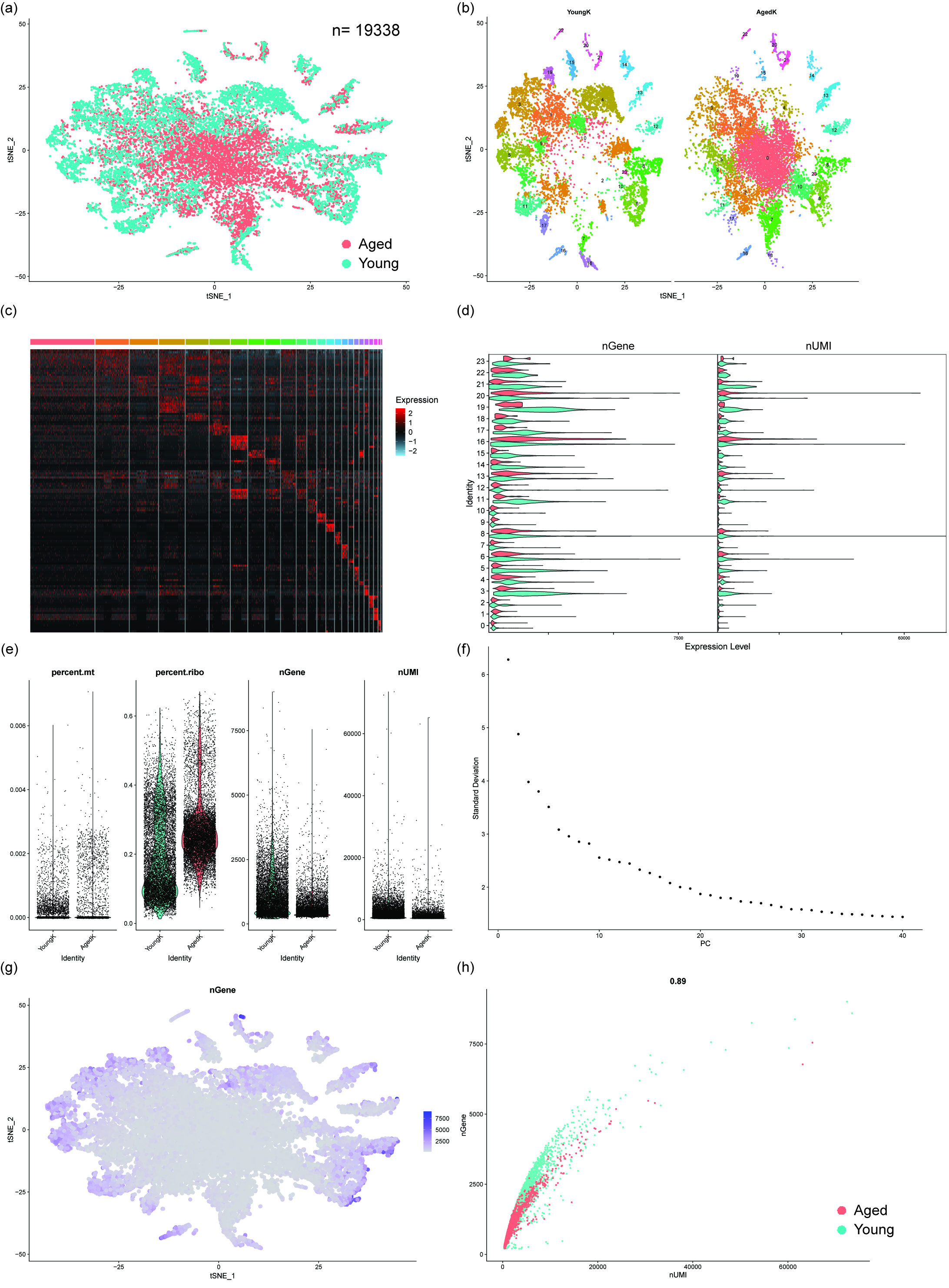

Supplement: Supplementary file 1 — Figures S1–S7. [file ACEL-23-e14251-s002.zip › acel14251-sup-0003-FigureS3.tif]

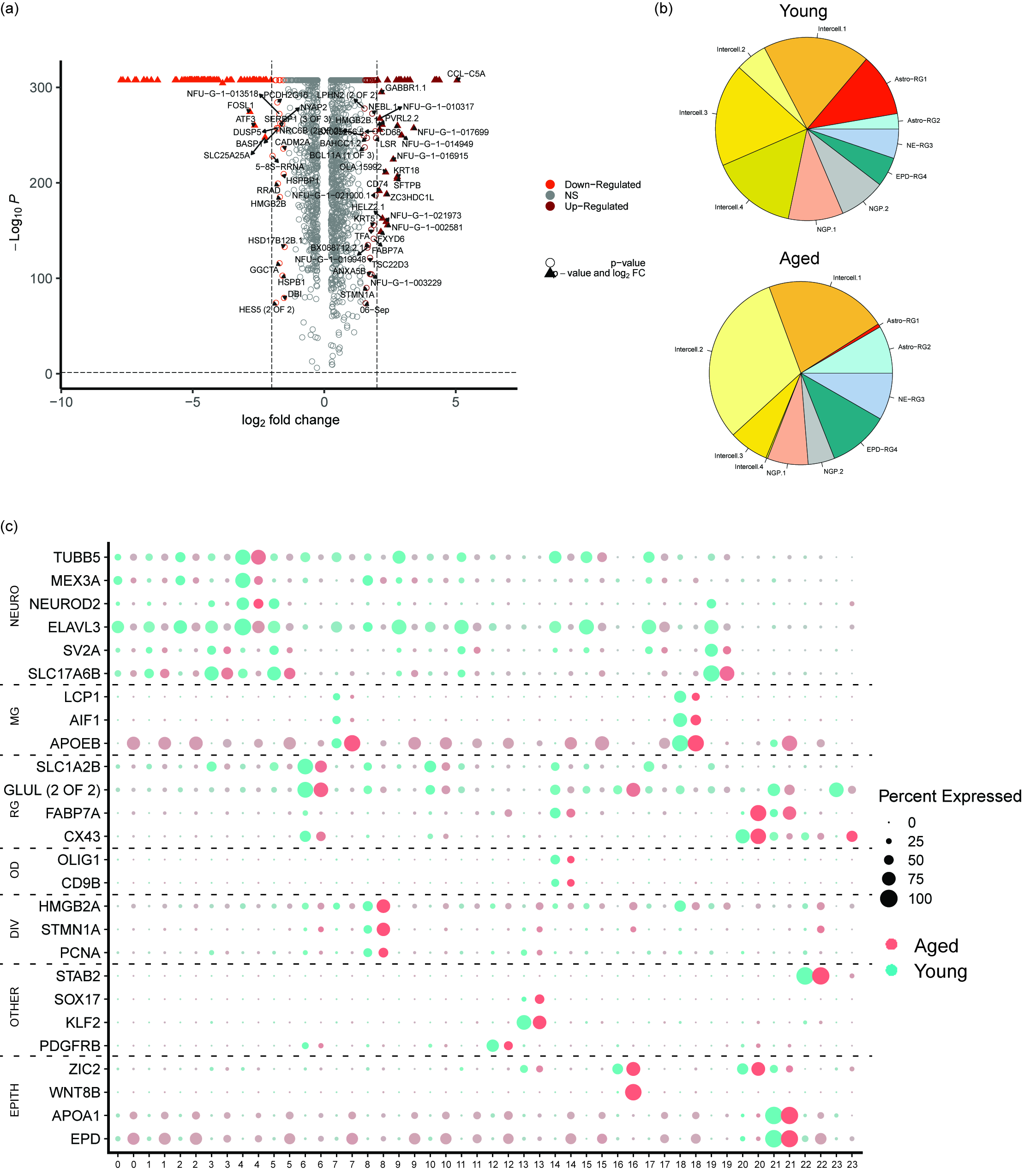

Supplement: Supplementary file 1 — Figures S1–S7. [file ACEL-23-e14251-s002.zip › acel14251-sup-0004-FigureS4.tif]

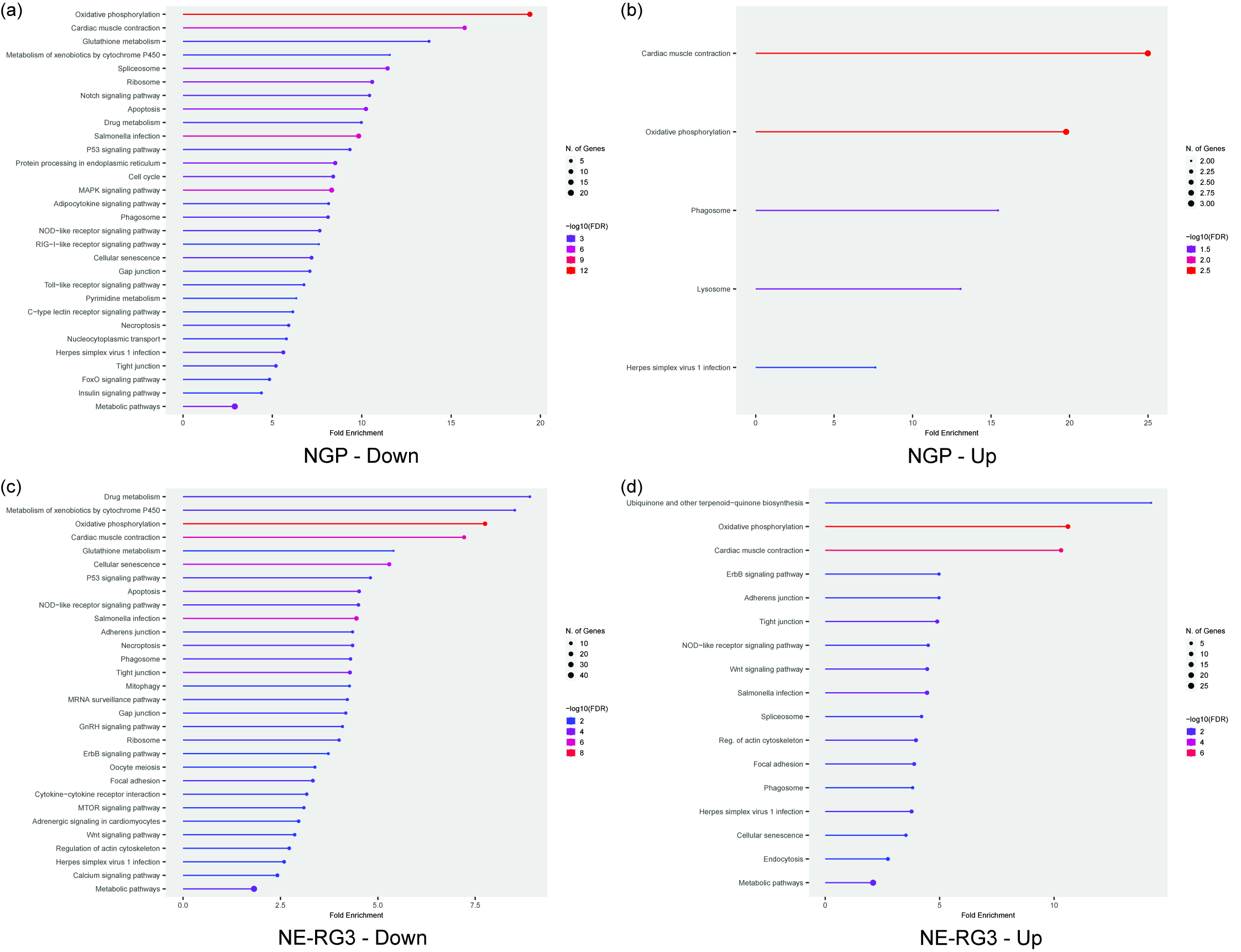

Supplement: Supplementary file 1 — Figures S1–S7. [file ACEL-23-e14251-s002.zip › acel14251-sup-0005-FigureS5.tif]

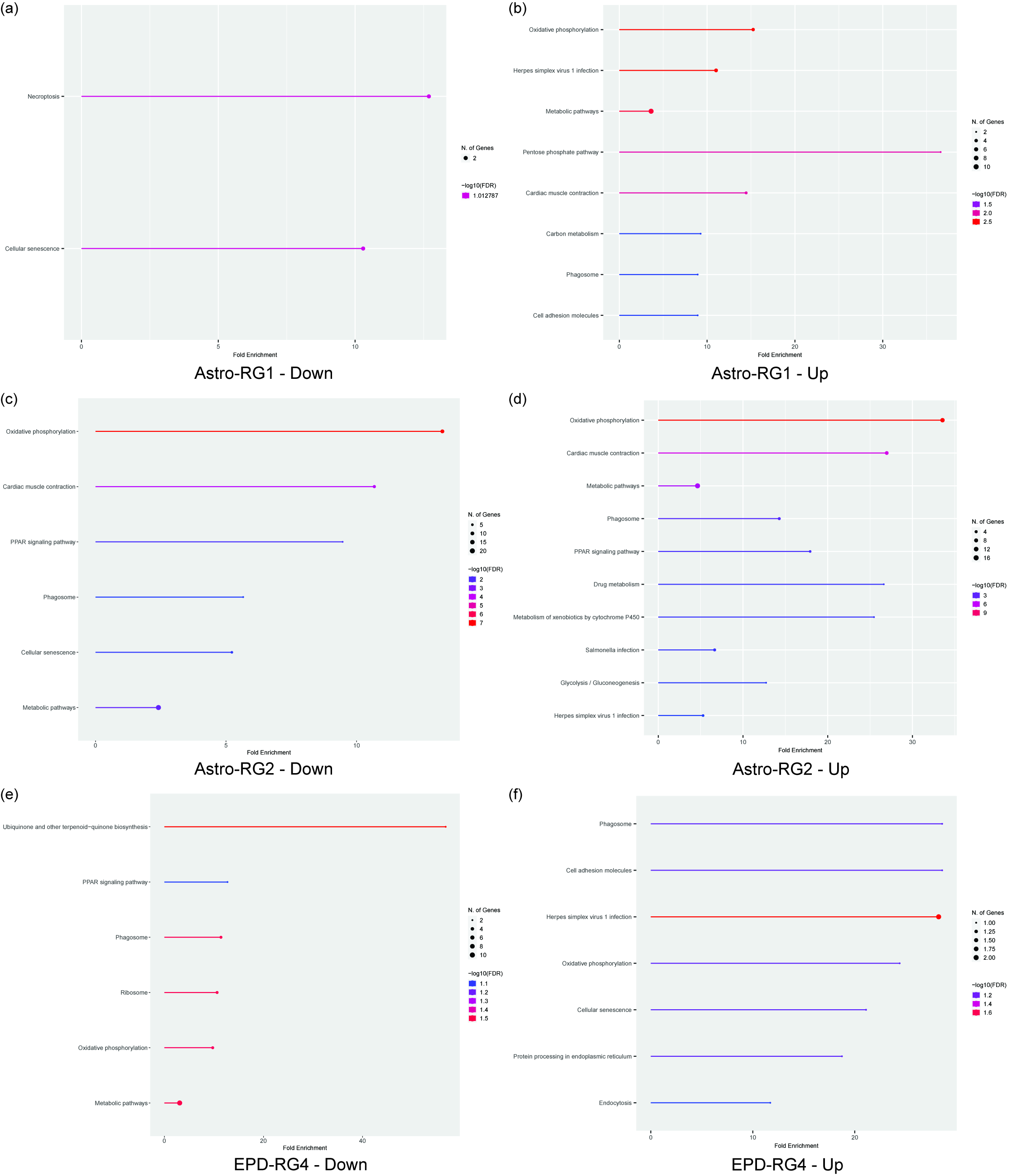

Supplement: Supplementary file 1 — Figures S1–S7. [file ACEL-23-e14251-s002.zip › acel14251-sup-0006-FigureS6.tif]

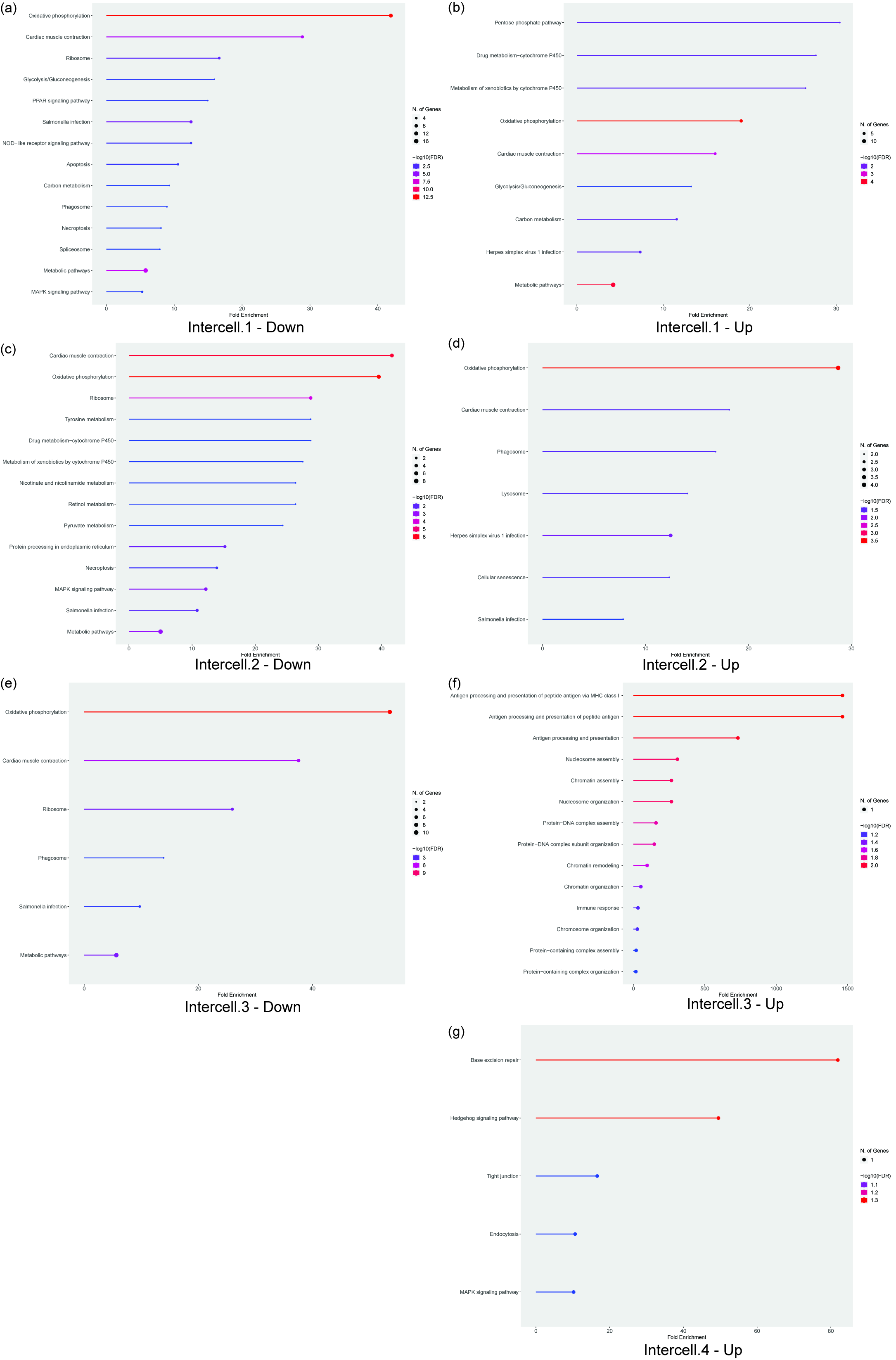

Supplement: Supplementary file 1 — Figures S1–S7. [file ACEL-23-e14251-s002.zip › acel14251-sup-0008-FigureS7.tif]
